# Targeting the CAF-GDF15 axis attenuates AKT-mediated mitochondrial rewiring and chemoradiation resistance in esophageal adenocarcinoma

**DOI:** 10.1186/s13046-026-03776-6

**Published:** 2026-07-17

**Authors:** Ningbo Fan, Pinwei Deng, Zhefang Wang, Feng Ju, Lisa Raatz, Oscar Velazquez Camacho, Axel M. Hillmer, Margarete Odenthal, Phuong-Hien Nguyen, Zicheng Lyu, Felix Popp, Seung-Hun Chon, Maarten F. Bijlsma, Romy Ros, Hanneke W. M. van Laarhoven, Alexander Quaas, Jörg Wischhusen, Yue Zhao, Christiane J. Bruns

**Affiliations:** 1 Department of General, Visceral, Thoracic and Transplantation Surgery, University Hospital of Cologne, Cologne, 50937 Germany; 2 Institute of Pathology, University of Cologne, Faculty of Medicine and University Hospital of Cologne, Cologne, 50937 Germany; 3 Center for Molecular Medicine Cologne, University of Cologne, Cologne, 50931 Germany; 4 Department I of Internal Medicine, University of Cologne, Faculty of Medicine and University Hospital Cologne, Center for Integrated Oncology Aachen Bonn Cologne Duesseldorf, Center for Molecular Medicine Cologne, Cologne, 50931 Germany; 5 Laboratory for Experimental Oncology and Radiobiology, Center for Experimental and Molecular Medicine, Amsterdam UMC, University of Amsterdam, RDC Adore, Amsterdam, The Netherlands; 6 Cancer Center Amsterdam, Imaging and Biomarkers, Amsterdam, the Netherlands; 7 Department of Medical Oncology, Amsterdam UMC, University of Amsterdam, Amsterdam, The Netherlands; 8 Department of Thoracic Oncology, State Key Laboratory of Oncology in South China, Collaborative Innovation Center for Cancer Medicine, Sun Yat-Sen University Cancer Center, Guangzhou, 510060 China; 9 Department for Obstetrics & Gynaecology, University Hospital Würzburg, Section for Experimental Tumor Immunology, Josef-Schneider-Str. 4, Würzburg, 97080 Germany

**Keywords:** Esophageal adenocarcinoma, Cancer-associated fibroblasts, Mitochondrion, GDF15, Organoids, Treatment resistance

## Abstract

**Background:**

Therapeutic resistance in esophageal adenocarcinoma (EAC) remains poorly understood at the level of tumor-stroma interactions. We investigated whether cancer-associated fibroblast (CAF)-derived signaling contributes to chemoradiation resistance and adverse clinical outcomes in EAC, with a focus on the functional role of GDF15 within the CAF-secreted program.

**Methods:**

Serum GDF15 levels were analyzed in EAC patients before and after neoadjuvant chemoradiotherapy according to the CROSS regimen, with prognostic relevance validated in an independent cohort and public datasets. Primary EAC CAFs, EAC cell lines, and patient-derived organoids (PDOs) were used to model tumor-stroma crosstalk in 2D and 3D co-culture systems. Compartment-specific transcriptomic and proteomic profiling identified CAF-regulated pathways. Genetic depletion and antibody-mediated neutralization of GDF15 were used to assess its functional contribution, followed by evaluation of chemoradiation sensitivity, mitochondrial function, oxidative phosphorylation dependency, and AKT pathway activation.

**Results:**

Serum GDF15 levels increased significantly following CROSS treatment, and elevated post-treatment GDF15 independently predicted poor overall survival in EAC patients. CAFs enhanced resistance to chemotherapy and radiotherapy in EAC cells and PDOs, accompanied by increased GDF15 secretion. Compartment-specific transcriptomic analysis and ELISA supported CAFs as a major source of inducible GDF15 during tumor-stroma interaction. Genetic depletion of GDF15 reduced treatment resistance, whereas recombinant GDF15 partially restored chemoresistance in GDF15-depleted models. GDF15 neutralization attenuated CAF-associated cisplatin tolerance. Mechanistically, GDF15 contributed to AKT activation and mitochondrial respiratory adaptation, including enhanced oxidative phosphorylation. Pharmacological attenuation of oxidative phosphorylation further sensitized EAC cells to cisplatin, particularly under CAF co-culture conditions.

**Conclusions:**

Our study identifies CAF-derived GDF15 as a clinically relevant and functionally targetable component of a broader CAF-secreted resistance program in EAC. GDF15 contributes to AKT-associated mitochondrial adaptation and tumor cell tolerance to chemoradiation, while elevated post-CROSS serum GDF15 serves as a prognostic biomarker. These findings support further investigation of GDF15-directed and mitochondria-targeted strategies to overcome CAF-associated treatment resistance in esophageal adenocarcinoma.

**Supplementary Information:**

The online version contains supplementary material available at https://doi.org/10.1186/s13046-026-03776-6.

## Background

Esophageal cancer (EC) represents a significant global health burden, with approximately 604,100 new cases and 544,076 cancer-related deaths in 2020 [1]. Esophageal adenocarcinoma (EAC) and esophageal squamous cell carcinoma (ESCC) are the two main subtypes of EC. An increasing trend in EAC incidence, accompanied by a decrease in ESCC incidence, has been observed in Western countries over the past decades [2, 3]. Surgery-based neoadjuvant chemotherapy (NCT) or neoadjuvant chemoradiotherapy (NCRT) are standard treatment strategies in the management of locally advanced EC [4, 5]. Despite the increasing advancements in the multidisciplinary management of EC patients, the prognosis of esophageal cancer remains poor, with a 5-year survival rate of around 20% [2, 3]. The unfavorable prognosis of EAC underscores the need for a deeper comprehension of tumor progression and the formulation of advanced therapeutic strategies.

The tumor microenvironment (TME) has been widely acknowledged as a crucial factor dynamically regulating tumor growth and progression [6, 7]. TME shields tumor cells from treatment by providing a physical barrier and a supportive niche, exerting paracrine effects, inducing tumor cell quiescence, enriching cancer stemness, and modulating metabolism and immune profiles [8, 9]. As the main components within the TME, cancer-associated fibroblasts (CAFs) play a crucial role in reorganizing the TME under intrinsic and extrinsic stresses, thereby facilitating tumor metastasis, therapeutic resistance, tumor dormancy, and reactivation [10, 11]. CAFs are increasingly recognized as a heterogeneous stromal population comprising distinct functional states, although an EAC-specific CAF subtyping framework remains to be fully established. CAF-mediated tumor resistances to radiotherapy, targeted therapy, and immunotherapy have been reported in various gastrointestinal (GI) cancers, including EC [12, 13].

Growth differentiation factor 15 (GDF15) is a divergent member of the TGF-β superfamily [14, 15]. The function of GDF15 in cancer has garnered significant research attention in recent decades. It has been reported that elevated GDF15 serum concentrations independently predict all-cause mortality in patients with various diseases including solid tumors [16, 17]. The combination of a neutralizing anti-GDF15 antibody and an anti-PD-1 antibody shows durable responses in lung, liver and urothelial cancer patients resistant to prior anti-PD-1/PD-L1 therapy, as demonstrated in the first-in-human phase 1/2a GDFATHER trial [18]. In EAC, tissue GDF15 expression is significantly elevated in patients with EAC compared to those with Barrett’s esophagus (BE) and healthy controls [19]. Similarly, serum GDF15 is increased in EAC patients compared to healthy controls, and a high GDF15 concentration is associated with poor survival in patients with EAC and adenocarcinoma of gastroesophageal junction (AGEJ) [19, 20]. To date, there is limited literature discussing the molecular functions of GDF15 in EAC patients, and its specific role in the interaction between CAFs and EAC cells in the TME remains unknown.

Patient-derived organoids (PDOs) are primary cells cultured in a three-dimensional (3D) system that closely mimics human development and organ regeneration in vitro [21, 22]. Derived from primary tumor tissues, PDOs recapitulate transcriptome and proteome profiles, metabolic functions, and microscale tissue architecture of the original organs [21]. This advanced model provides valuable insights for drug development and personalized cancer treatment, offering a more accurate representation of organ biology and enhancing our understanding of disease mechanisms [23, 24]. Owing to their potential in recreating complex in vitro tumor microenvironments and accelerating the development of anticancer therapeutics, PDO models may also reduce the need for animal experiments [25–27].

In this study, we aim to explore the impact of EAC CAFs on treatment resistance in EAC cells, seeking to uncover potential mechanisms underlying this interaction. Using patient-derived primary tumor tissues, we generated EAC CAFs and tumor PDOs, enabling exploration through both 2D and 3D co-culture systems. We identify that GDF15 enhances mitochondrial function in EAC cells, contributes to treatment resistance, and predicts patient survival after CROSS treatment. Our findings underscore the significant impact of EAC CAFs on treatment resistance in EAC tumor cells, partly through the secretion of GDF15, and the consequential activation of the AKT pathway. These insights highlight the potential of GDF15 as a crucial prognostic marker and a promising therapeutic target in EAC.

## Methods

### Patient samples

Patient samples were obtained from the surgical biobank of the University Hospital of Cologne and from Amsterdam University Medical Centers (Amsterdam UMC), with institutional ethical approval and written informed consent.

Primary CAFs were established from tumor specimens of five treatment-naïve EAC patients. Two CAF lines (TBE60 and TBE63) were derived from diagnostic endoscopic biopsies, and three (CAF2304, CAF2765, and CAF3095) from surgical resection specimens. All patients had histologically confirmed esophageal adenocarcinoma and had not received systemic therapy at the time of sample collection. Clinical characteristics are summarized in Supplementary Table S1.

For serum analyses, a consecutive cohort of 55 EAC patients treated with neoadjuvant chemoradiotherapy according to the CROSS regimen at the University Hospital of Cologne was retrospectively identified. Paired serum samples were collected before and after treatment and used for GDF15 measurement and survival analysis. Patient characteristics were comparable between GDF15-low and GDF15-high groups (Supplementary Table S2).

Patients were stratified into 'GDF15-high' and 'GDF15-low' groups based on optimal cut-off points determined by X-tile software (version 3.6.1; Yale University, New Haven, CT). This method defines thresholds by identifying the values that maximize the log-rank X^2^ statistic, providing the greatest statistical separation in clinical outcomes. The calculated optimal cut-offs were 883 pg/mL for pre-CROSS treatment and 1542 pg/mL for post-CROSS treatment [28].

An independent cohort of 23 EAC patients from Amsterdam UMC with paired pre- and post-CROSS serum samples was included as an external validation cohort for serum GDF15 analyses.

### Cell culture

#### EAC tumor cell lines

Two human EAC cell lines, OE19 and OE33, were used in this study. Both cell lines were obtained from commercial sources as listed in the Key Resources Table. Cells were cultured in RPMI 1640 medium supplemented with 10% fetal bovine serum (FBS) and 1% penicillin–streptomycin (P/S). Cells were maintained at 37 °C in a humidified incubator with 5% CO₂.

#### CAFs cell line

Five primary EAC CAF lines (TBE60, TBE63, CAF2304, CAF2765, and CAF3095) were established in-house (Bruns/Zhao group, Surgical Laboratory, Department of General, Visceral, Thorax and Transplant Surgery, University Hospital of Cologne). CAFs were cultured in Advanced DMEM/F-12 medium supplemented with 10% FBS, 1% P/S, and 1% L-glutamine. Clinical characteristics of the corresponding patients are summarized in Supplementary Table S1.

#### PDOs culture

Three EAC PDO lines (PDO2304, PDO2765, and PDO3095), each paired with their corresponding CAFs, were used in this study. PDOs were established in-house from primary EAC tumor tissues obtained at surgical resection. PDOs were embedded in Matrigel domes and cultured in EAC PDO medium consisting of Wnt-3a-conditioned medium, R-spondin1-conditioned medium, N-2 supplement, B-27 supplement, 1 mM N-acetyl-L-cysteine, 0.5 µM CHIR-99021, 250 ng/mL recombinant human EGF, 100 ng/mL FGF10, 0.5 µM A83-01, 1 µM SB202190, 0.5 µM Gastrin I, 20 µM Nicotinamide, 100 ng/mL Noggin, 10 µM Y-27632, 10 µM Gentamicin, 100 µg/mL Normocin, and 1% P/S. PDO culture medium was refreshed every 4–6 days [29].

PDOs were maintained either by mechanical passaging or by enzymatic dissociation into single cells using 0.25% trypsin–EDTA, depending on experimental requirements. Single-cell-derived PDOs were used for experiments requiring controlled cell number and size quantification, whereas mechanically passaged PDOs were used for histological analyses and long-term expansion.

#### Establishment of primary CAFs

Primary CAFs were isolated from EAC tumor tissues obtained by endoscopic biopsy or surgical resection. Tissue specimens were washed, minced into small fragments, and seeded onto collagen type I-coated six-well plates (1:20 dilution in DPBS). Tissues were cultured in CAF medium supplemented with 0.6% Amphotericin B and 0.2% Normocin during the first week. Medium was partially replaced after 4–5 days and fully refreshed every 3–5 days thereafter. Migrating fibroblasts were expanded and cryopreserved for subsequent experiments.

#### Establishment of EAC PDOs

Fresh EAC tumor tissues were washed with DPBS, mechanically minced, and enzymatically digested using a buffer containing Dispase, Collagenase IV, Amphotericin B, and Y-27632. Additional dissociation was performed using 0.25% trypsin–EDTA. Cell suspensions were filtered through a 100-µm strainer, embedded in Matrigel, and cultured in EAC PDO medium. Established PDOs were expanded and cryopreserved for future use.

#### Co-culture systems

For 2D co-culture experiments, CAFs were seeded in six-well plates, while EAC tumor cells were seeded onto 0.4-µm pore size transwell inserts, allowing paracrine interaction without direct cell–cell contact.

For 3D co-culture experiments, two systems were employed. In the dome-based co-culture system, paired CAFs were seeded in separate Matrigel domes within the same well and cultured in shared PDO medium, enabling paracrine interaction while preventing direct contact and facilitating CAF-free PDO harvesting. In the transwell-based 3D co-culture system, CAFs were seeded in the lower chamber, while PDOs embedded in Matrigel were cultured on 0.4-µm pore size transwell inserts.

#### Quality control

All cell lines and PDO cultures were routinely tested for mycoplasma contamination using the MycoStrip detection kit according to the manufacturer’s instructions.

### Cell proliferation assay

Cells were seeded in 24-well plates and incubated overnight. The medium was replaced the next day, with subsequent medium refreshment every 2–4 days. The plates were fixed with 4% ROTI Histofix on days 0, 2, 4, and 6. After fixation, the plates were stained with 0.05% Crystal violet solution and imaged for visual results. To quantify the staining, the Crystal violet particles were dissolved in an equal volume of 10% acetic acid. Subsequently, 100 µL of each dissolved crystal violet solution was transferred to a 96-well plate, and absorbance was measured at 570 nm. All assays were performed in triplicates. Relative cell proliferation was calculated by normalizing the measurements to day 0 values using GraphPad Prism.

### Cell viability assay

To evaluate cell drug sensitivity and viability, the MTT assay was performed for 2D cell lines, while the luminescent cell viability assay was employed for EAC PDOs.

In the MTT assay, cells were seeded into a 96-well plate at consistent density and incubated overnight. Subsequently, the cells were exposed to increasing concentrations of chemotherapeutic drugs for 48 h. After treatment, the cells were incubated with 5 mg/mL MTT solution at 37 °C for 3.5–4 h. Following incubation, the MTT solution was discarded, and MTT solvent was added to each well to dissolve the MTT formazan crystals. Absorbance at 570 nm was measured, and the collected data were subjected to analysis.

For luminescent cell viability analysis, EAC PDOs were harvested and enzymatically digested into single cells. The single cells were subsequently mixed with 1:10 dilution of Matrigel matrix and PDO medium, and evenly distributed into a 96-well plate. When small spheres emerged after 3–5 days of seeding, increasing concentrations of chemotherapeutic drugs were introduced to the plates. Following a 48-h incubation period, cell viability was assessed using the CellTiter-Glo luminescent cell viability assay in accordance with the manufacturer's instructions. Luminescence signals were recorded for data analysis.

Data analysis was performed using GraphPad Prism. Each assay was conducted in triplicates.

### CCK8 cell viability assay

For the GDF15 neutralization and low-dose oligomycin A experiments, cell viability was assessed using the Cell Counting Kit-8 (CCK8) assay according to the manufacturer’s instructions. Briefly, OE33 cells were seeded into 96-well plates at a consistent density and allowed to attach overnight. Cells were then treated with conditioned medium, cisplatin, GDF15-neutralizing antibody, IgG1 isotype control antibody, oligomycin A, or the indicated combinations for 48 h. At the end of treatment, CCK8 reagent was added directly to each well and incubated at 37 °C for 4–6 h until sufficient color development was achieved. Absorbance was measured at 450 nm using a microplate reader. Relative cell viability was calculated by normalizing absorbance values to the corresponding control group. Data were analyzed using GraphPad Prism.

### Apoptosis assay

The cells were treated either with cisplatin or exposed to ionizing radiation using the BIOBEAM GM gamma irradiation device. After 48 h, the cells were harvested and suspended in Annexin V binding buffer containing Annexin V-APC and DAPI staining dye. The suspension was incubated for 20 min. Subsequently, flow cytometry analysis was performed using the Attune™ NxT Flow Cytometer, and the data were analyzed using FlowJo software.

### GDF15-neutralizing antibody experiments

GDF15 neutralization experiments were performed using an anti-human GDF15-neutralizing antibody (α-h-GDF-15, 0297; produced by Evitria, Schlieren, Switzerland), with an IgG1 control antibody used as the corresponding control. Antibodies were used at a final concentration of 10 μg/mL unless otherwise indicated.

For conditioned medium experiments, TBE63 CAFs were cultured until confluent, followed by medium replacement. After 24 h, the supernatant was collected as TBE63-conditioned medium. OE33 cells were treated with TBE63-conditioned medium together with GDF15-neutralizing antibody, IgG1 isotype control antibody, cisplatin, or the indicated combinations for 48 h. Cell viability was then assessed by CCK8 assay.

For transwell co-culture experiments, OE33 cells were co-cultured with TBE63 CAFs in a transwell system. After establishment of the co-culture, GDF15-neutralizing antibody or IgG1 isotype control antibody was added and incubated for 48 h. OE33 cells were then collected and treated with cisplatin for an additional 48 h, followed by CCK8-based cell viability analysis. For apoptosis analysis, cells were collected after the indicated treatments and stained with Annexin V-APC and DAPI before being subjected to flow cytometric analysis.

### Quantitative Real-time PCR (qPCR) analysis

Cultured cells or EAC tissue samples were subjected to RNA extraction using TRI reagent. The High-Capacity cDNA Reverse Transcription Kit was employed for reverse transcription following the manufacturer's instructions. The relative expression levels of target mRNAs were determined using the Fast SYBR green master mix and measured by the QuantStudio 7 Flex Real-Time PCR System. Relative gene expression from qPCR experiments was analyzed using the 2^−ΔΔCt^ method. The primers utilized in this study are listed in Table S4.

### Western blot

For protein extraction, cells were harvested and lysed on ice using RIPA buffer. The lysates were subjected to the Bioruptor Pico sonication system for 10 cycles at 4 °C to facilitate protein degradation. Protein samples were collected by centrifugation at 12,000 × g for 10 min at 4 °C to remove cell debris. Protein concentrations were determined using the BCA protein assay kit. The protein samples were then prepared and denatured in NuPAGE LDS sample buffer and heated at 70 °C for 10 min.

Twenty micrograms of protein were loaded onto SDS-PAGE gel and subsequently transferred to a PVDF membrane using the Trans-Blot Turbo transfer system. The transferred membranes were blocked in 1 × Roti-Block for 1 h. Following the blocking step, the membranes were incubated overnight at 4 °C with specific primary antibodies. After thorough washing, the blots were probed with secondary antibodies specific to the primary antibodies for 1 h. Protein bands were visualized using the SuperSignal West Pico PLUS Chemiluminescent Substrate and detected with the ChemoStar ECL Imager.

### Immunofluorescence staining and immunohistochemistry staining

For immunofluorescence (IF) staining, cells were fixed with 4% ROTI Histofix for 15 min, followed by permeabilization with 0.2% Triton X-100 in DPBS for 15 min. To block non-specific binding, cells were incubated with normal serum block for 40 min. Subsequently, the cells were incubated overnight at 4 °C with primary antibodies. After washing with DPBS, the cells were incubated with fluorochrome-conjugated secondary antibodies for 1 h. Nuclei were counterstained with DAPI, and fluorescence images were captured using the Olympus IX83 inverted microscope.

As to immunohistochemistry (IHC) staining, paraffin embedded EAC PDO slides were deparaffinized by incubating at 60 °C for 1 h, followed by sequential washes with xylene and graded ethanol solutions. Antigen retrieval was performed by heating the slides in a pH6.0 citrate buffer using the PT-Module. After cooling, endogenous peroxidase was inactivated by incubating the sections with 0.3% hydrogen peroxide solution in methanol for 20 min. Subsequently, the slides were incubated overnight at 4 °C with primary antibodies diluted in antibody diluent reagent solution. After washing with 1X TBS buffer, the slides were incubated with a secondary antibody conjugated to horseradish peroxidase (HRP) for 45 min. Detection of the antibody signal was achieved using EnVision system-HRP. Finally, the slides were counterstained with Mayer’s hematoxylin solution, followed by dehydration and mounting. The stained slides were visualized using an inverted light microscope.

### Enzyme-linked immunosorbent assay (ELISA)

The concentration of GDF15 in patient serum samples and cell culture media was analyzed using the human GDF-15 Quantikine ELISA Kit following the manufacturer's instructions. Initially, samples were centrifuged and filtered to eliminate particulates and further diluted with calibrator diluent at a ratio of 1/4 or 1/5. The samples and standards were added to the wells and allowed to incubate for two hours. Then, the wells were washed and the GDF15 conjugate was introduced for an additional incubation of one hour. After another washing step, the substrate solution was added to initiate a colorimetric reaction, which was stopped using the stop solution after 30 min. The absorbance of each well was measured using a microplate reader at 450 nm. GDF15 concentration was calculated using a linear regression fit based on the standard curve. Three replicates were measured for each sample.

### Plasmid construction and lentiviral transduction

For RNA interference, plasmids expressing the short hairpin RNA (shRNA) targeting GDF15 were constructed using the pLKO.1 puro vector. The shRNA sequences targeting GDF15 were designed using the Genetic Perturbation Platform (GPP). The forward and reverse oligonucleotides containing the shRNA sequences were synthesized and annealed. The pLKO.1 puro vector was linearized, and the annealed shRNA oligonucleotides were ligated into the vector through Agel and EcoRI. The resulting ligation mixtures were transformed into competent cells, and positive colonies were selected by ampicillin and confirmed by DNA sequencing.

Stable cell lines expressing the shRNA sequence were generated through lentiviral transduction. HEK293T cells were transfected with transfer plasmid using a 2nd generation packaging system through polyethylenimine (PEI) in a mass ratio of 1:3 DNA/PEI. The medium was replaced 24 h post-transfection, and the supernatant containing the virus was collected and filtered through a 0.45 µm strainer at 48 h and 72 h. The virus-containing supernatant was mixed in a 1:1 ratio with fresh medium supplemented with 8 µg/mL polybrene and used for cell transduction. Puromycin selection was initiated 48–72 h post-transduction, and the selective medium was refreshed every 2–3 days and maintained for 1 week.

### Mitochondrial stress test

Cell mitochondrial stress test was performed using the Agilent Seahorse XF Cell Mito Stress Test Kit following the manufacturer's instructions. Cells were seeded in XF cell culture plates and incubated until reaching the desired confluency. The assay medium was prepared by supplementing the XF DMEM medium with 10 mM Glucose, 1 mM Pyruvate, and 2 mM Glutamine. The cells were washed and incubated with assay medium in a non-CO_2_ incubator at 37 °C for 1 h prior to assay. The assay plate was prepared by adding injection solutions including Oligomycin, Carbonyl cyanide-p-trifluoromethoxy phenylhydrazone (FCCP), and Rotenone/Antimycin A (Rot/AA). The assay plate was then inserted into the Seahorse XFe96 Analyzer, and the instrument sequentially injected 2.5 µM oligomycin, 1 µM FCCP, and 0.5 µM Rot/AA while simultaneously measuring the real-time oxygen consumption rate (OCR) and extracellular acidification rate (ECAR) of the cells. Four to six replicates were measured for each sample. Data were analyzed by Wave software and visualized using GraphPad Prism.

### Measurement of mitochondrial membrane potential by TMRE staining

Mitochondrial membrane potential was assessed using tetramethylrhodamine ethyl ester (TMRE) staining followed by flow cytometry. Briefly, OE33 cells were treated with cisplatin, GDF15-neutralizing antibody, IgG1 isotype control antibody, or the indicated combinations for 48 h. After treatment, the culture medium was removed, and cells were gently washed once with pre-warmed PBS. Cells were then incubated with fresh complete medium containing 200 nM TMRE for 20 min at 37 °C in the dark. After staining, the TMRE-containing medium was removed, and cells were trypsinized, collected, and resuspended in PBS. A live/dead viability dye was added before flow cytometric analysis to exclude dead cells.

Flow cytometry was performed using a CytoFLEX LX flow cytometer (Beckman Coulter Life Sciences). TMRE fluorescence was measured in the PE channel, and mitochondrial membrane potential was analyzed in live cells after exclusion of debris and dead cells. Data were analyzed using FlowJo software, and TMRE median fluorescence intensity was used to quantify mitochondrial membrane potential.

### Next-generation RNA sequencing (RNA-seq) sample preparation and data analysis

The EAC cell line OE33 was subjected to a transwell co-culture with two CAF cell lines, TBE60 and TBE63, for a duration of seven days. Parallel culturing of OE33, TBE60, and TBE63 was also performed as a control. Triplicate samples from each experimental group were subjected to RNA extraction using the previously described method. Following our previous study [29], mRNA-seq libraries were prepared for sequencing using the QuantSeq 3' mRNA-Seq Library Prep Kit FWD for Illumina according to the low-input protocol. Subsequently, constructed libraries were sequenced on the HiSeq 4000 system by 1 × 50 bases.

STAR software [30] was used to align the high-throughput reads to the human genome (Homo sapiens GRCh38). The aligned reads were counted with HTSeq, and differential gene expression analysis was performed using the DESeq2 R package [31] in R software. P values were adjusted for multiple testing using the Benjamini–Hochberg false discovery rate (FDR) method. Differentially expressed genes were defined by an adjusted *p*-value < 0.05 and an absolute log2 fold change ≥ 1 unless otherwise indicated.

### Proteomics sample preparation and data analysis

OE33 sh-GDF15 cell line was supplemented with 50 ng/mL human recombinant GDF15 protein (rGDF15) for seven days, along with parallel culturing of OE33 sh-NT and OE33 sh-GDF15 cell lines. Protein samples were prepared according to the instructions from Proteomics Core Facility Cologne with materials provided. Cells were lysed with 8 M urea/50 mM triethylammonium bicarbonate (TEAB) buffer and subjected to the Bioruptor Pico sonication system for chromatin degradation. Protein concentrations were measured using the BCA method and 50 µg protein per sample was collected. Samples were subsequently incubated with 5 mM dithiothreitol (DTT) for one hour, followed by 40 mM chloroacetamide (CAA) in the dark for 30 min, and then incubated with Lysyl endopeptidase (Lys-C) at a ratio of 1/75 for 4 h. After the incubation, the samples were diluted with 50 mM TEAB to achieve a final urea concentration of less than 2 M and incubated with trypsin at a 1/75 enzyme-to-substrate ratio overnight. Enzymatic digestion was stopped by acidifying the samples with formic acid to a final concentration of 1%, and samples were further purified using SDB-RP StageTips prior to measurement. Samples were analyzed by LC–MS/MS on a Q-Exactive Plus mass spectrometer coupled to an Easy nanoLC 1000 system. The raw data were processed by MaxQuant [32] using standard settings with label-free quantification (LFQ) activated. The match-between-runs function was enabled between replicates. Protein analysis was performed and visualized based on LFQ using the R package ‘DEP’ in R software [33]. Protein differential abundance analysis was performed based on LFQ intensities using the DEP R package. Multiple testing correction was performed using the Benjamini–Hochberg FDR method, and significantly regulated proteins were defined by an adjusted* p*-value < 0.05 unless otherwise indicated.

### Public database

Public databases were utilized for data extraction and analysis to assess the correlation between GDF15 expression and clinical significance in EAC patients. The TCGA (Esophageal Adenocarcinoma, PanCancer Atlas) database was accessed and downloaded from cBioPortal [34]. A total of 182 patients’ mRNA expression data with matched clinical information were extracted, including 95 patients with ESCC and 87 patients with EAC. The GSE26886 and GSE92396 datasets were obtained from the NCBI GEO database [35] for gene expression analysis. GSE26886 database provides gene expression profiling of 20 Barrett's esophagus patients, 21 EAC patients, nine ESCC patients, and 19 patients with normal esophageal squamous epithelium [36]. GSE92396 database contains nine paired samples of EAC tumor and adjacent normal tissue.

### Statistics

All in vitro experiments were performed as at least three independent biological replicates. For in vitro cell-based assays, data are presented as mean ± standard deviation (SD). The number of replicates is specified in each figure legend. Statistical comparisons between two groups were performed using unpaired or paired Student’s t-test as appropriate. Comparisons among multiple groups were performed using one-way ANOVA followed by the indicated multiple-comparison test. Parametric tests were only applied to approximately normally distributed data; variability is shown as mean ± SD from independent biological replicates. Survival analysis was performed using the Kaplan–Meier method, and statistical differences were evaluated using the log-rank test. Univariate and multivariate Cox regression analyses were performed to identify risk factors associated with overall survival in EAC patients. For RNA-seq and proteomics analyses, multiple comparisons were corrected using adjusted p-values or FDR-based correction as described above. A two-sided *p*-value < 0.05 was considered statistically significant unless otherwise stated. Data were analyzed using GraphPad Prism software, IBM SPSS Statistics software, and R software.

## Results

### Serum and tissue GDF15 are elevated in EAC and high post-CROSS serum GDF15 predicts poor prognosis

**Fig. 1:**
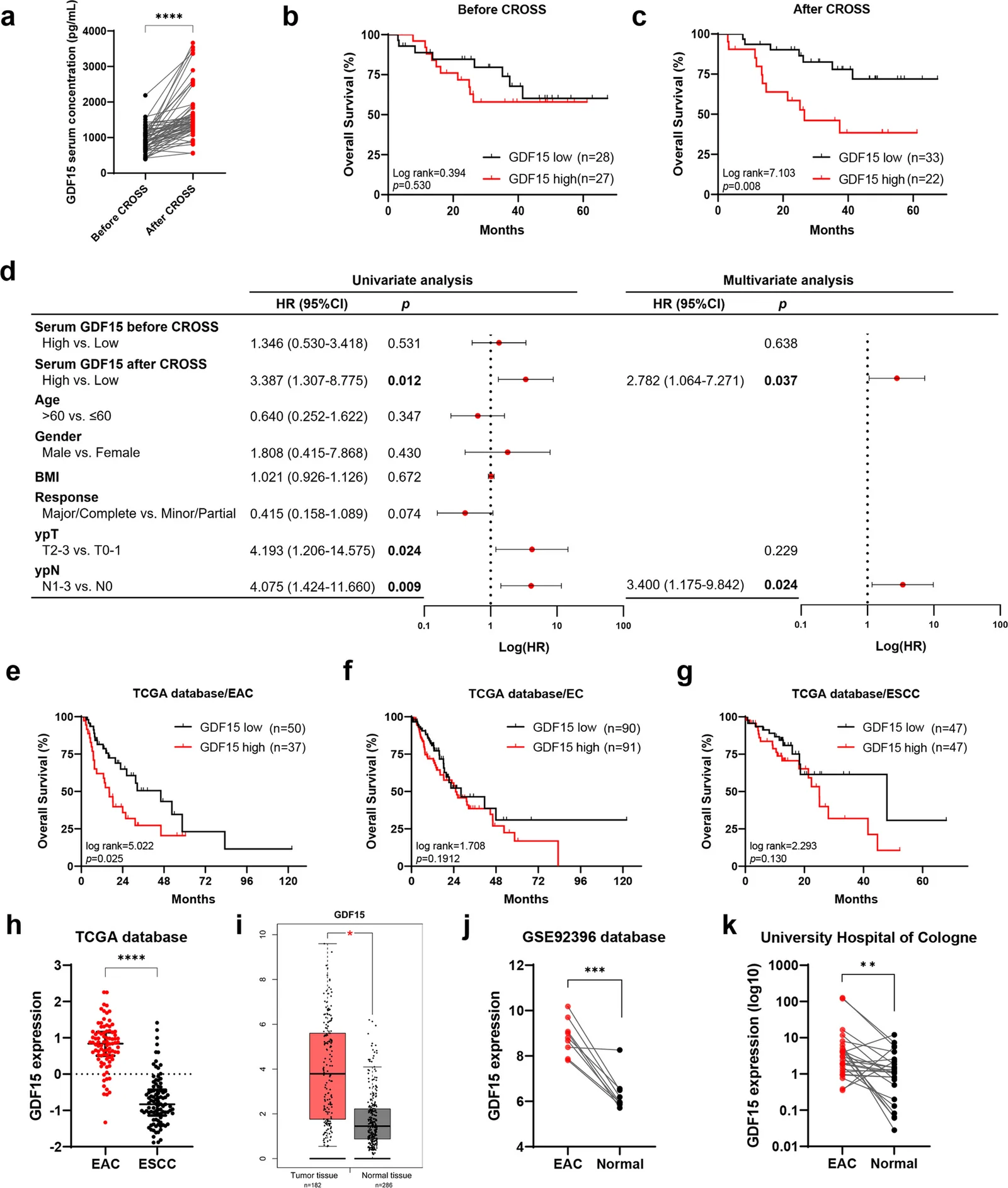
Serum and tissue GDF15 are elevated in EAC and high post-CROSS serum GDF15 predicts poor prognosis. **a** Paired serum GDF15 concentration before and after the CROSS treatment in 55 EAC patients from University Hospital of Cologne. Serum GDF15 is significantly higher after the CROSS treatment. Paired t-test, *n* = 55, ****p < 0.0001. **b**-**c** Survival analysis of GDF15 high group and GDF15 low group that are divided according to the serum GDF15 concentrations before and after the CROSS treatment. Before the CROSS treatment, there is no significant difference between the serum GDF15 high and low groups regarding OS (log-rank = 0.394, n = 55, *p* = 0.530). After the CROSS treatment, high levels of serum GDF15 are significantly associated with reduced OS in EAC patients (log-rank = 7.103, *n* = 55, *p* = 0.008). **d** Univariate and multivariate Cox regression analyses of prognostic factors for overall survival in EAC patients. Bold text indicates a significant difference. **e**. High levels of GDF15 expression are significantly associated with reduced overall survival in EAC patients from the TCGA database. Log-rank = 5.022, *n* = 87, *p* = 0.025. **f**-**g**. High levels of GDF15 expression are associated with a trend towards poor prognosis in both EC patients and the ESCC subpopulation from the TCGA database, but without statistical significance (log-rank = 1.708, *n* = 181, *p* = 0.1912 for EC patients, and log-rank = 2.293, *n* = 94, *p* = 0.130 for ESCC subpopulation). **h** GDF15 expression is significantly higher in EAC patients as compared to ESCC patients in the TCGA database. Unpaired t-test, *n* = 87 for EAC, *n* = 94 for ESCC, ****p < 0.0001. **i** GDF15 expression is significantly higher in TCGA tumor tissues compared to TCGA normal and Genotype-Tissue Expression (GTEx) normal tissues. Unpaired t-test, *n* = 182 for tumor tissues, *n* = 286 for normal tissues, *p < 0.05. **j** GDF15 expression is significantly higher in EAC tumor tissues as compared to adjacent normal tissues in the GSE92396 database. Paired t-test, *n* = 9, ***p < 0.001. **k** GDF15 expression is significantly higher in EAC tumor tissues as compared to adjacent normal tissues in 25 paired EAC tumor and normal tissues from the University Hospital of Cologne. Paired t-test, *n* = 25, **p < 0.01

The CROSS regimen, which consists of weekly administration of carboplatin and paclitaxel for five weeks combined with concurrent radiotherapy (total dose 41.4 Gy), represents a standard neoadjuvant chemoradiotherapy (NCRT) approach for the treatment of locally advanced esophageal cancer [4, 5]. To investigate the clinical relevance of GDF15, we retrospectively reviewed the surgical biobank of the University Hospital of Cologne and identified a consecutive cohort of 55 EAC patients with paired serum samples collected before and after CROSS treatment between January 2017 and December 2020. Serum GDF15 concentrations were measured in these paired samples, revealing a significant increase following CROSS treatment (938.5 ± 341.7 pg/mL vs. 1672 ± 742.1 pg/mL, *p* < 0.0001; Fig. 1a).

Patients were subsequently stratified into GDF15-low and GDF15-high groups before and after CROSS treatment. Detailed patient characteristics were summarized in Table S2 . Prior to treatment, 28 patients were classified as GDF15-low (683.8 ± 154.6 pg/mL) and 27 as GDF15-high (1202.7 ± 274.4 pg/mL). After CROSS treatment, 33 patients belonged to the GDF15-low group (1237.1 ± 260.5 pg/mL), while 22 patients were classified as GDF15-high (2325.0 ± 754.1 pg/mL). No significant differences were observed between the GDF15-low and GDF15-high groups with respect to age, sex, body mass index (BMI), response to CROSS treatment, ypT stage, or ypN stage, either before or after treatment.

Kaplan–Meier survival analysis demonstrated no significant difference in OS between the GDF15-low and GDF15-high groups prior to CROSS treatment (Log-rank = 0.394, *p* = 0.530; Fig. 1b). In contrast, elevated serum GDF15 levels after CROSS treatment were significantly associated with inferior OS in EAC patients (Log-rank = 7.103, *p* = 0.008; Fig. 1c).

These findings were independently validated in an external cohort of 23 EAC patients from Amsterdam UMC. Consistently, serum GDF15 concentrations significantly increased after CROSS treatment (1410 ± 550 pg/mL vs. 1759 ± 465 pg/mL, *p* ≤ 0.001; Figure S1a). Although high post-treatment GDF15 levels showed a trend toward poorer overall survival, the association did not reach statistical significance (Log-rank = 0.605, *p* = 0.437; Figure S1b-c), likely due to the limited sample size of the validation cohort.

Univariate Cox regression analysis identified high post-CROSS serum GDF15 concentration (HR = 3.387, 95% CI: 1.307–8.775, *p* = 0.012), higher ypT stage (HR = 4.193, 95% CI: 1.206–14.575, *p* = 0.024), and higher ypN stage (HR = 4.075, 95% CI: 1.424–11.660, *p* = 0.009) as significant predictors of poor OS (Fig. 1d). Importantly, multivariate Cox regression analysis confirmed that elevated serum GDF15 concentration after CROSS treatment remained an independent prognostic factor for OS in EAC patients (HR = 2.782, 95% CI: 1.064–7.271, *p* = 0.037; Fig. 1d).

Consistent with these clinical findings, analysis of The Cancer Genome Atlas (TCGA) dataset demonstrated that high GDF15 expression was significantly associated with reduced OS in the EAC subpopulation (Log-rank = 5.022, *p* = 0.025; Fig. 1e), but not in the overall esophageal cancer cohort (Log-rank = 1.708, *p* = 0.191; Fig. 1f) or in the ESCC subpopulation (Log-rank = 2.293, *p* = 0.130; Fig. 1g). Furthermore, GDF15 expression was significantly higher in EAC compared to ESCC tissues in the TCGA cohort (Fig. 1h). Analysis using GEPIA2 [37] further revealed that GDF15 expression was significantly elevated in TCGA tumor tissues compared with both TCGA normal and GTEx normal tissues (Fig. 1i).

Finally, paired tissue analyses demonstrated that GDF15 expression was significantly upregulated in EAC tumor tissues compared with adjacent normal tissues, as observed in the GSE92396 dataset (Fig. 1j) and in our in-house cohort of 25 paired EAC samples (Fig. 1k; Table S3).

Together, these findings indicate that GDF15 has clear clinical relevance in EAC, with elevated post-CROSS GDF15 levels being closely associated with poor overall survival, supporting its use as a prognostic marker in EAC patients.

### EAC CAFs promote EAC cell proliferation and treatment resistance in vitro* and* GDF15 is involved in the crosstalk between EAC cells and EAC CAFs

A total of five primary EAC CAF cell lines were used in this study, with TBE63 and TBE60 derived from endoscopic biopsies, while CAF2304, CAF2765 and CAF3095 were obtained from surgical specimens. The clinical information of EAC patients for EAC CAFs included in this study were summarized in Table S1. We validated EAC CAFs using fibroblast markers (αSMA, IL-6, PDGFRα, vimentin) and confirmed their distinction from epithelial cells with the marker Pan-CK. The immunofluorescence (IF) staining showed that all five CAFs were αSMA positive but Pan-CK negative, while the EAC cell line OE33, serving as a control, expressed Pan-CK but not αSMA (Figure S2a). Besides, we compared the mRNA expression levels of fibroblast-related markers in our CAFs and EAC tumor cell lines OE33 and OE19. The results showed that αSMA, IL-6, PDGFRα, and vimentin were expressed heterogeneously in CAF cell lines but not expressed in tumor cell lines (Figure S2b). To further characterize CAF heterogeneity, we analyzed the expression of representative marker genes for myCAF, iCAF, and vCAF subset. This analysis showed that TBE60 and TBE63 displayed heterogeneous CAF-state marker profiles, including myofibroblastic and inflammatory signatures, whereas OE33 cells showed negligible expression of these stromal programs (Figure S2c).

**Fig. 2:**
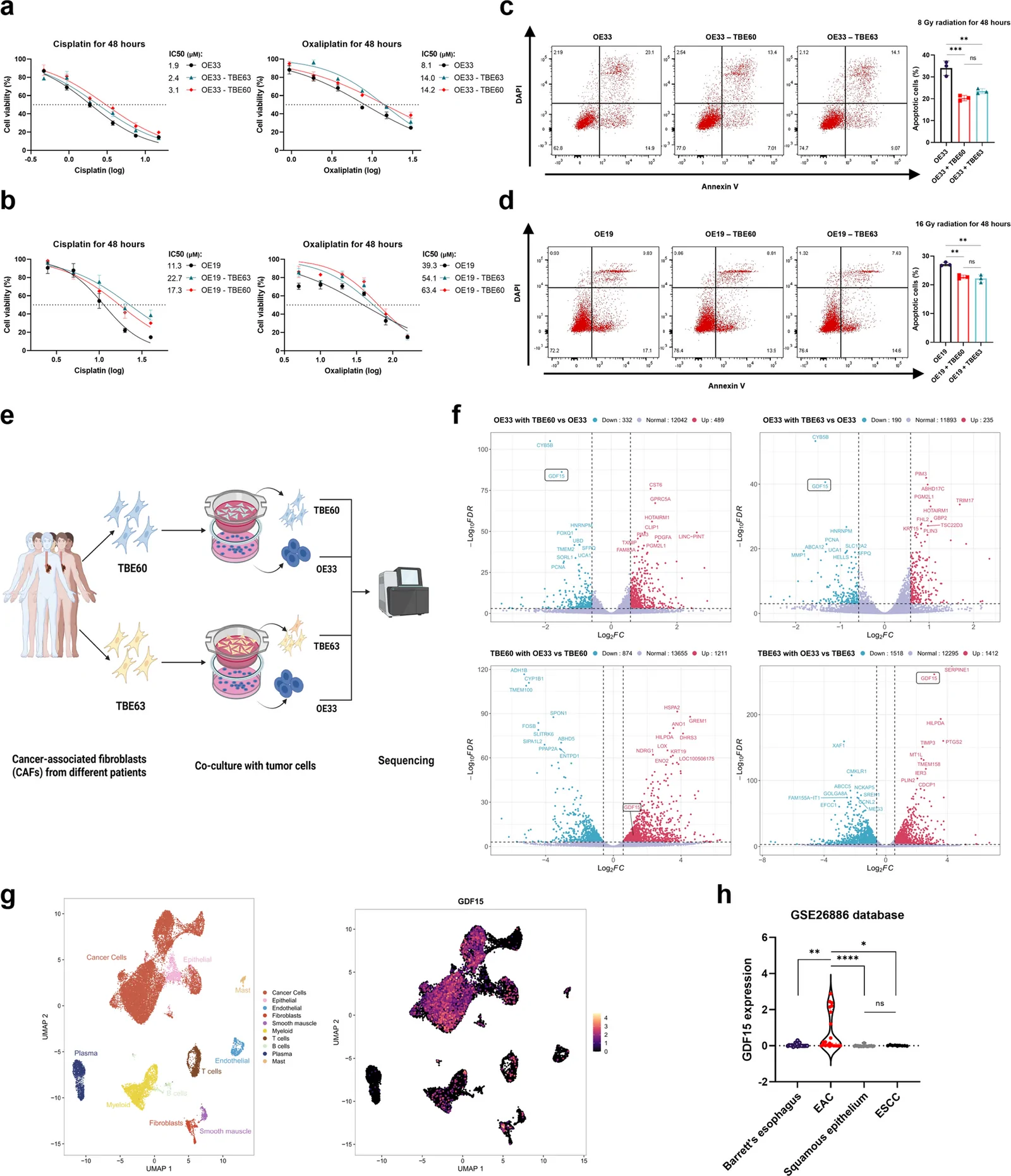
EAC CAFs promote EAC cell treatment resistance in vitro* and* GDF15 is involved in the crosstalk between EAC cells and EAC CAFs. **a**-**b** Cell viability assay shows that OE33/OE19 are more resistant to cisplatin and oxaliplatin treatments after co-culture with two different EAC CAFs TBE60 and TBE63. Data are represented as mean ± SD, *n* = 3. **c**-**d** Apoptosis assay shows that after co-culture with two different EAC CAFs TBE60 and TBE63, OE33/OE19 have fewer apoptotic populations 48 h after exposure to 8 Gy/16 Gy ionizing radiation. Data are represented as mean ± SD, *n* = 3. Unpaired t-test, ns: *p* > 0.05, **p < 0.01, ***p < 0.001. **e** Sequencing workflow for patient-derived CAFs and tumor cell (OE33) co-cultures. **f** Volcano plots showing dysregulated genes in OE33, TBE60, and TBE63 after seven days of transwell co-culture. The plot highlights several dysregulated genes, including GDF15, with an opposite expression pattern in OE33 cells and CAFs. **g** UMAP plots depicting the major cell types identified by single-cell RNA sequencing of tissues from four EAC patients (University Hospital of Cologne). GDF15 is highly expressed in both cancer cells and fibroblasts. **h** GDF15 expression is significantly higher in the EAC tissues as compared to ESCC, Barrett’s esophagus, and squamous epithelium in the GSE26886 database. Unpaired t-test, *n* = 19 for squamous epithelium, *n* = 20 for Barrett’s esophagus, *n* = 21 for EAC, and *n* = 8 for ESCC, ns: *p* > 0.05, *p < 0.05, **p < 0.01, ****p < 0.0001

To explore the interaction between EAC cells and EAC CAFs in vitro, a transwell co-culture set-up was used. Following a seven-day co-culture period, EAC cells OE33 and OE19 showed enhanced proliferation ability after co-culture with two different EAC CAFs (TBE60 and TBE63) (Figure S3a-b). Besides, OE33 and OE19 were more resistant to cisplatin and oxaliplatin treatment in vitro (Fig. 2a-b).

We further validated enhanced EAC cell radioresistance and chemoresistance using flow cytometry-based apoptosis assays. Consistently, OE33 and OE19 showed significantly decreased apoptotic cell populations after co-culture with EAC CAFs when exposed to radiotherapy treatment (8 Gy and 16 Gy, respectively) and cisplatin treatment (3 μM and 30 μM, respectively) for 48 h (Fig. 2c-d and Figure S3b-c). Taken together, these data suggest that EAC CAFs promote EAC cell proliferation and treatment resistance in vitro.

To further understand the potential molecular mechanism of the crosstalk between EAC cells and EAC CAFs, we performed a transcriptomic analysis of the co-culture setup. OE33 was co-cultured with TBE60 and TBE63 for seven days, and both cells, with and without co-culture, were subjected to high-throughput RNA sequencing (Fig. 2e). Transcriptomic analysis [29–31] identified several dysregulated genes, among which GDF15 stood out due to its opposite expression pattern in OE33 and two CAF cell lines after co-culture. GDF15 expression was upregulated in CAFs but downregulated in OE33, suggesting a possible dynamic interaction between CAFs and tumor cells (Fig. 2f). We validated GDF15 expression at single-cell level in four EAC patients from a previously published dataset of our group [38]. The results showed that GDF15 was highly expressed in both fibroblasts and EAC tumor cells, highlighting its potential relevance in CAF-mediated disease pathogenesis (Fig. 2g). Next, we investigated the clinical significance of GDF15 in patients with esophageal cancer using public databases [34, 35]. The analysis of the GSE26886 dataset [36] showed that GDF15 expression was significantly higher in EAC tissues as compared to ESCC, Barrett's esophagus, and squamous epithelium (Fig. 2h).

In summary, transcriptomic analysis of in vitro EAC tumor-stroma co-culture identified GDF15 as a key mediator exhibiting opposite regulation in EAC cells and CAFs. GDF15 is upregulated in EAC tissues and is associated with poor clinical outcome specifically in EAC patients, highlighting its potential role in CAF-driven tumor progression and treatment resistance.

### GDF15 depletion results in reduced treatment resistance in EAC cells and reduced EAC CAF-mediated tumor treatment resistance in vitro

To further investigate the role of GDF15 in the interaction between EAC cells and CAFs, we first quantified GDF15 concentrations in the supernatants of EAC cell monocultures, CAF monocultures, and transwell co-cultures. In a total culture volume of 3 mL, supernatants were collected 48 h after co-culture for ELISA-based GDF15 detection. No GDF15 was detected in EAC medium alone, whereas OE19, OE33, TBE60, and TBE63 monocultures secreted different levels of GDF15. Basal GDF15 secretion was relatively low in OE19 cells (75.22 ± 21.17 pg/mL) and higher in OE33 cells (750.24 ± 19.85 pg/mL). Among CAF monocultures, TBE63 secreted higher levels of GDF15 than TBE60 (828.99 ± 79.35 pg/mL vs. 218.06 ± 32.75 pg/mL), indicating CAF-line-dependent heterogeneity in basal GDF15 secretion.

**Fig. 3:**
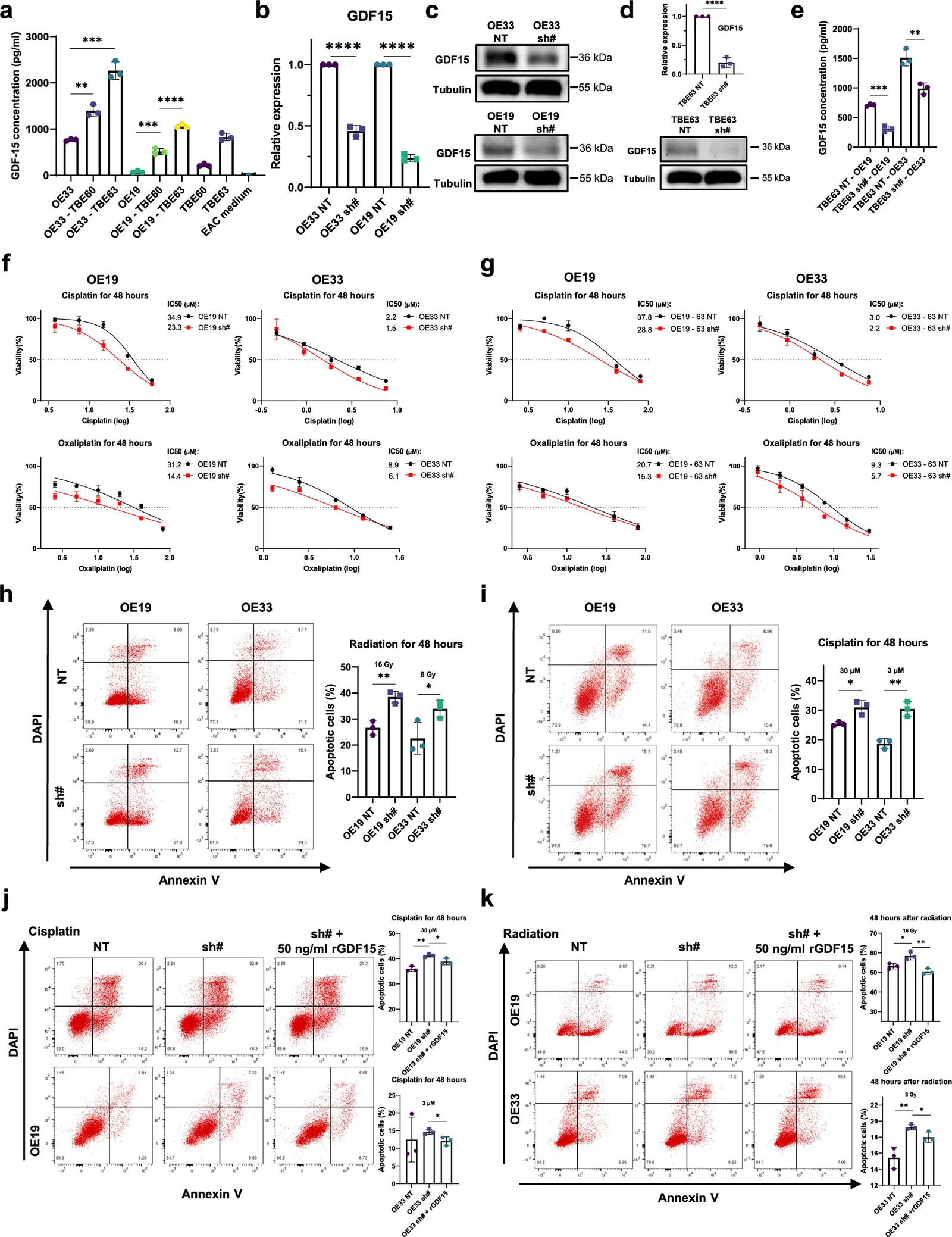
GDF15 depletion results in reduced treatment resistance in EAC cells and reduced EAC CAF-mediated treatment resistance in vitro. **a** GDF15 concentrations are significantly higher in the co-culture medium in both OE33 and OE19 cells. Data are represented as mean ± SD, *n* = 3. Unpaired t-test, **p < 0.01, ***p < 0.001, ****p < 0.0001. **b**-**c** Validation of GDF15 knockdown (sh#) and control (NT) tumor cells in qPCR and western blot. Data are represented as mean ± SD, *n* = 3. Unpaired t-test, ****p < 0.0001. **d** Validation of GDF15 knockdown and control CAF cell line TBE63 in qPCR and western blot. Unpaired t-test, *n* = 3, ****p < 0.0001. **e** Transwell co-culture setup of co-culturing EAC cell lines with GDF15-depleted/control TBE63 cells. GDF15 concentrations are significantly decreased in OE19/OE33-TBE63 GDF15 knockdown co-culture medium. Data are represented as mean ± SD, *n* = 3. Unpaired t-test, **p < 0.01, ***p < 0.001. **f** Cell viability assay shows increased sensitivity to cisplatin and oxaliplatin treatments after GDF15 knockdown in OE19 and OE33 cells. Data are represented as mean ± SD, *n* = 3. **g** Cell viability assay shows increased sensitivity to oxaliplatin and cisplatin treatment in OE19 and OE33 cells after co-culture with GDF15-depleted TBE63. Data are represented as mean ± SD, *n* = 3. **h** Apoptosis assay shows increased apoptotic populations in OE19 and OE33 GDF15 knockdown cells 48 h after exposure to ionizing radiation (16 Gy and 8 Gy, respectively). Data are represented as mean ± SD, *n* = 3. Unpaired t-test, *p < 0.05, **p < 0.01. **i** Apoptosis assay shows increased apoptotic populations in OE19 and OE33 GDF15 knockdown cells after 48 h cisplatin treatment (30 µM and 3 µM, respectively). Data are represented as mean ± SD, *n* = 3. Unpaired t-test, *p < 0.05, **p < 0.01. **j**-**k** Rescue assays show that exogenous human rGDF15 treatment restores resistance to chemotherapy and ionizing radiation in GDF15-depleted OE19 and OE33 cells. The apoptosis assay is performed to determine cell sensitivity by calculating the apoptotic cell populations after the treatment. Data are represented as mean ± SD, *n* = 3. Unpaired t-test, *p < 0.05, **p < 0.01

Compared with monoculture conditions, GDF15 concentrations were further increased in all EAC cell-CAF co-culture supernatants. GDF15 levels reached 471.64 ± 80.74 pg/mL in OE19-TBE60 co-culture and 1066.40 ± 38.27 pg/mL in OE19-TBE63 co-culture. Similarly, OE33-TBE60 and OE33-TBE63 co-cultures showed increased GDF15 concentrations of 1360.77 ± 169.56 pg/mL and 2263.68 ± 188.90 pg/mL, respectively (Fig. 3a). The highest GDF15 concentration was observed in the OE33-TBE63 co-culture condition. These data indicate that GDF15 secretion is detectable in both EAC cells and CAFs but is substantially enhanced upon EAC cell-CAF interaction, supporting a dynamic regulation of GDF15 within the tumor-stroma ecosystem. Subsequently, we knocked down GDF15 in OE19 and OE33 cells using the short hairpin RNA system (Fig. 3b-c). No significant differences were observed regarding cell proliferation ability after GDF15 depletion in both OE33 and OE19 cells (Figure S4a-b).

To explore the function of GDF15 in CAF-mediated treatment resistance, we established a stable GDF15 knock down CAF cell line TBE63 (Fig. 3d). We performed transwell co-culture setup involving OE19 and OE33 cells with TBE63 GDF15-silenced cells and TBE63 control cells. GDF15 concentrations were significantly decreased in the OE19/OE33-TBE63 sh-GDF15 co-culture medium (308.62 ± 46.88 pg/mL, *p* = 0.0002 and 989.68 ± 94.34 pg/mL, *p* = 0.0059, respectively) as compared to OE19/OE33-TBE63 sh-NT groups (711.68 ± 21.30 pg/mL and 1512.12 ± 140.97 pg/mL, respectively) (Fig. 3e). These data suggest that CAFs contribute substantially to the extracellular GDF15 pool in the co-culture system, with its secretion being further amplified through EAC cell-CAF interaction.

Functionally, GDF15 depletion increased the sensitivity of both OE33 and OE19 cells to cisplatin and oxaliplatin treatment (Fig. 3f). In the transwell co-culture system, co-culture with GDF15-depleted TBE63 CAFs attenuated CAF-mediated chemoprotection in OE33 and OE19 cells (Fig. 3g). Moreover, GDF15 depletion increased radiation-induced apoptosis in EAC cells (Fig. 3h) and enhanced cisplatin-induced apoptosis after 48-h treatment (Fig. 3i). Rescue experiments using recombinant human GDF15 protein (rGDF15) partially restored the treatment resistance reduced by GDF15 knockdown in both OE19 and OE33 cells (Fig. 3j-k).

Taken together, GDF15 depletion resulted in reduced treatment resistance in EAC tumor cells and reduced EAC CAF-mediated tumor treatment resistance in the tumor microenvironment.

### CAF co-culture and rGDF15 supplementation increase treatment resistance in EAC PDOs

**Fig. 4:**
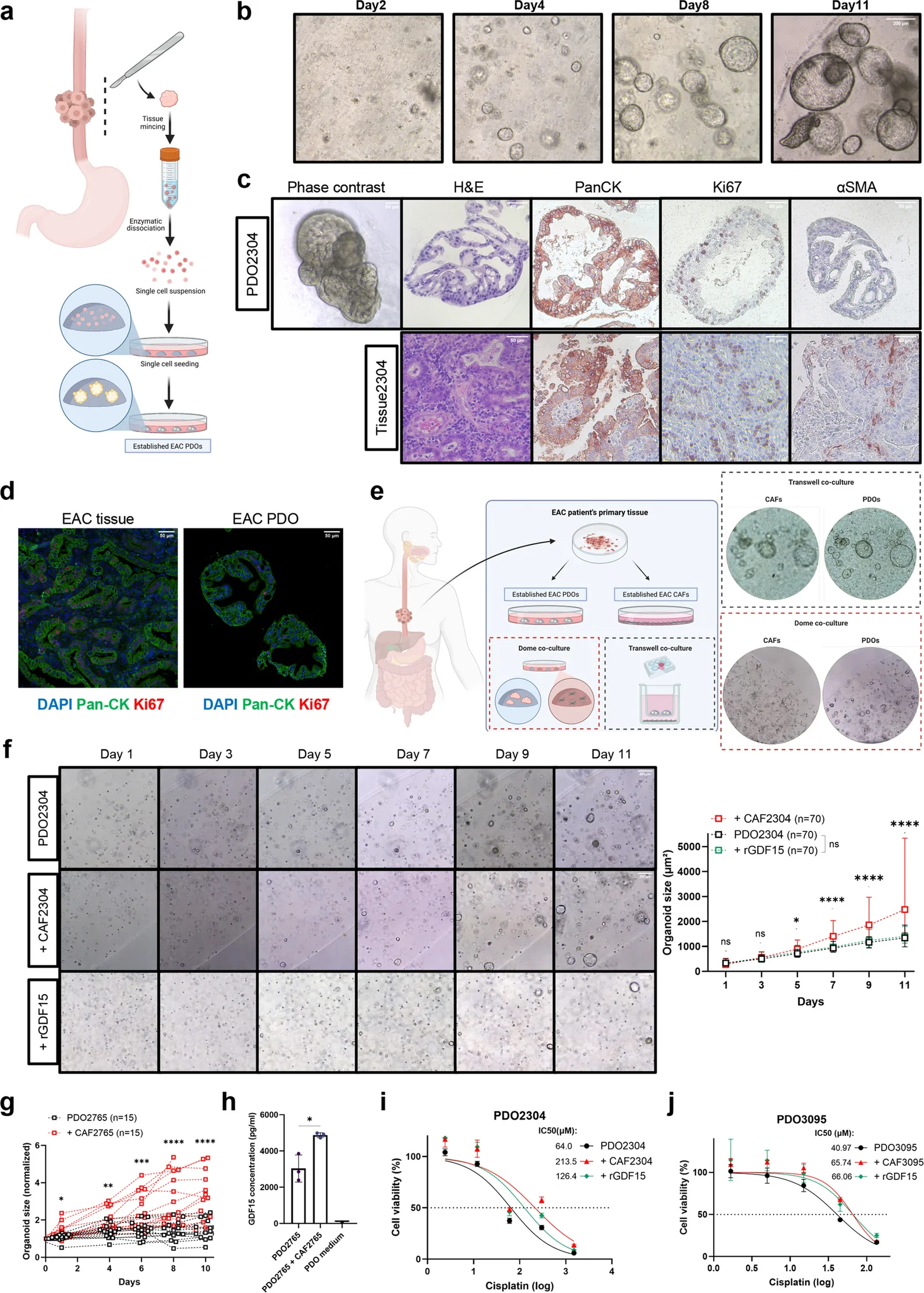
CAF co-culture and rGDF15 supplementation increase treatment resistance in EAC PDOs. **a** The workflow of establishing EAC PDOs from EAC patient primary tissues. **b** The phase-contrast images demonstrate the process of EAC organoids formation from single cells over the days. Scale bar: 200 µm. **c** Histological comparison of EAC PDOs and paired patient tissues. The figure includes a phase-contrast image of PDOs, as well as H&E staining and IHC staining for Pan-CK, Ki67, and αSMA in both PDOs and tissues. Scale bar: 50 µm. **d** The IF staining of epithelial marker Pan-CK (green), proliferation marker Ki67 (red) and DAPI (blue) in EAC PDOs and paired patient tissue. Scale bar: 50 µm. **e** The workflow of establishing EAC PDOs and EAC CAFs from same patient’s tissue for dome-based co-culture and transwell-based co-culture. **f** Growth kinetics of EAC PDOs with and without dome-based co-culture of paired CAFs and with human rGDF15 protein supplement. PDO sizes are individually tracked under an ECHO Rebel microscope, and the corresponding areas of PDOs are measured and digitized using ECHO pro software. The sizes of 70 individual PDOs from each group are measured, revealing a time-dependent increase that is significantly higher in the co-culture group than in the control group. There is no significant difference in size between the rGDF15 and control groups. Scale bar: 200 µm. Data are represented as mean ± SD, *n* = 70 per group. Paired t-test, ns: *p* > 0.05, *p < 0.05, ****p < 0.0001. **g** Growth kinetics of EAC PDOs with and without transwell co-cultured with paired CAFs. The sizes of 15 individual PDOs from each group are measured, revealing a time-dependent increase that is significantly higher in the co-culture group than in the control group. Data are represented individually, *n* = 15 per group. Paired t-test, *p < 0.05, **p < 0.01, ***p < 0.001, ****p < 0.0001. **h** GDF15 concentrations are significantly higher in the co-culture medium as compared to the control medium in the transwell co-culture system of EAC PDOs and paired CAFs. Data are represented as mean ± SD, *n* = 3. Unpaired t-test, *p < 0.05. **i**-**j**. Cisplatin dose–response analysis of two distinct EAC PDOs (PDO2304 and PDO3095) cultured alone, co-cultured with paired CAFs in the dome-based system or treated with human rGDF15. Cell viability was assessed after cisplatin treatment using a luminescent cell viability assay. Data are presented as mean ± SD, *n* = 3

We previously described the standard procedures for managing EAC PDOs in translational research [39]. The workflow for establishing EAC PDOs can be found in Fig. 4a. One typical example of organoid initiation is illustrated in Fig. 4b. We validated EAC PDOs by comparing their morphological and histological features with the paired tissues via IHC and IF staining (Fig. 4c-d). EAC PDOs shared similar expression patterns of Pan-CK and Ki67 with paired tumor tissues. We included αSMA as a negative control showing expression in paired tissues but not in PDOs, indicating the absence of contaminating fibroblasts in our tumor organoids.

To study the tumor-stroma interactions in a 3D model using the PDOs, we have established EAC PDOs with paired CAFs from the same patient’s tissue and performed a dome co-culture system as well as a transwell co-culture system. Example images of these two co-culture systems were shown in Fig. 4e. In line with the 2D culture results, paired CAFs promote EAC PDO proliferation in both dome-coculture system (Fig. 4f) and transwell co-culture system (Fig. 4g, Figure S5). For dome-based co-culture, 70 organoids from each co-culture group and control group were measured by size and individually tracked for 11 days. For transwell co-culture, 15 organoids from each group were monitored. We tested the GDF15 concentration in the transwell co-culture system. The supernatants were collected 72 h after the co-culture in a total of 4 mL culture medium. Consistently, GDF15 concentrations were significantly higher in the co-culture medium as compared to control (4876.38 ± 133.50 pg/mL vs. 3035.36 ± 747.91 pg/mL, *p* = 0.0137) (Fig. 4h).

In addition, EAC PDOs showed enhanced cisplatin resistance after being co-cultured with paired CAFs for two weeks in the dome co-culture system (Fig. 4i-j). We did not perform the drug sensitivity assay in the transwell co-culture system due to the limited number of PDO cells harvested from the chamber. Furthermore, the addition of human rGDF15 protein to the PDO culture medium did not increase PDO cell proliferation (Fig. 4f) but did enhance cisplatin resistance compared to the control (Fig. 4i-j).

Collectively, the 3D PDO model validated the findings from 2D cell line experiments that EAC CAFs promote EAC cell treatment resistance with the involvement of GDF15 as one of the contributing factors.

### GDF15 supports mitochondrial respiratory programs and mitochondrial adaptation in EAC cells

To explore the molecular mechanisms underlying the effects of GDF15 on EAC cells, we utilized proteomics analysis [32, 33] to investigate the alterations in the EAC cell proteome following GDF15 knockdown. Cells from OE33 sh-NT, OE33 sh-GDF15 and OE33 sh-GDF15 with 50 ng/mL rGDF15 treatment for seven days were collected. More than 4000 proteins were detected in each measured sample and distinct protein expression profiles were achieved among the experimental groups (Figure S6a-b).

**Fig. 5:**
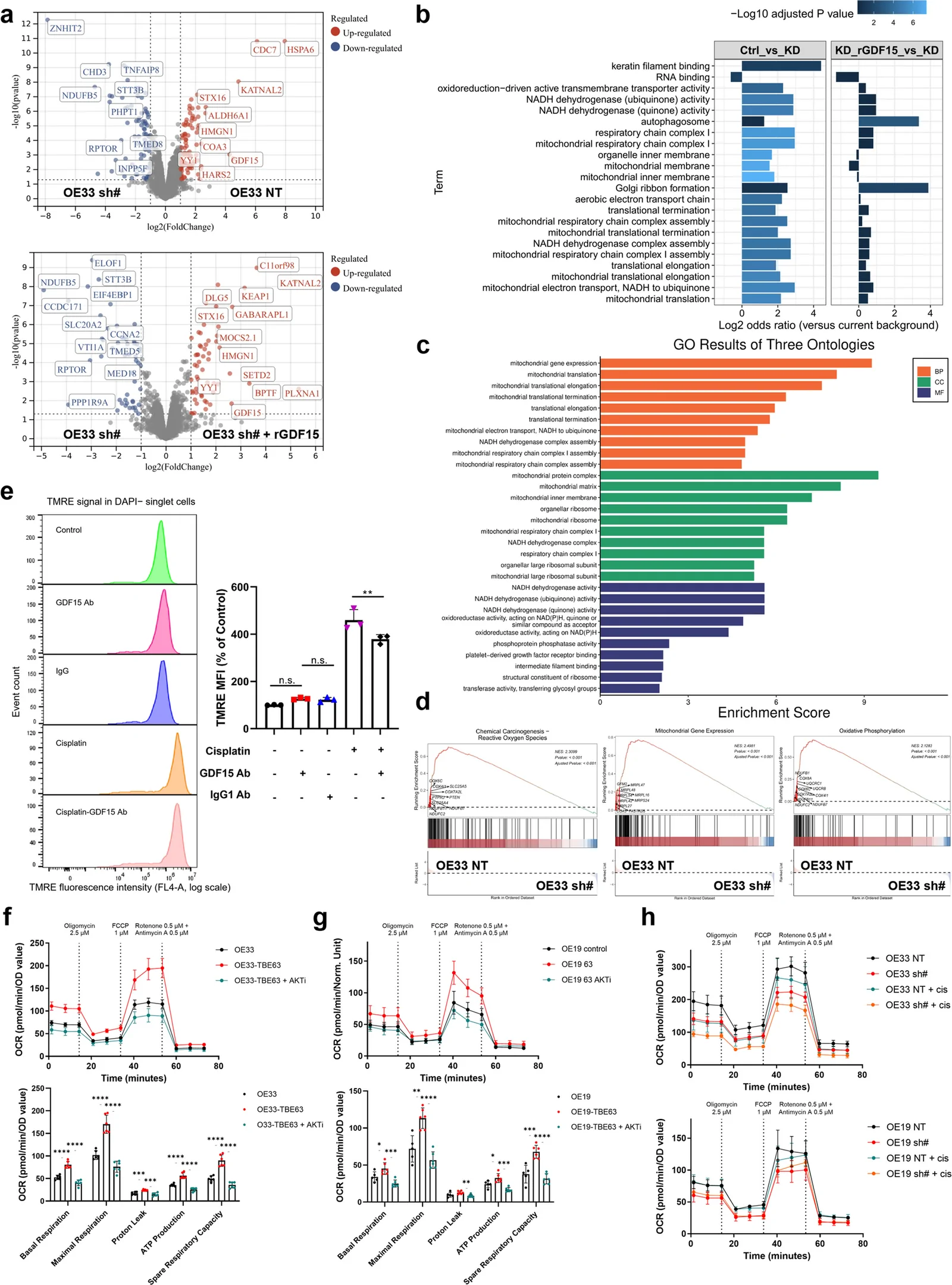
GDF15 depletion results in impaired mitochondrial function in EAC cells. **a** Volcano plots showing dysregulated proteins in OE33 sh-NT, OE33 sh-GDF15 and OE33 sh-GDF15 plus 50 ng/mL rGDF15 treatment for seven days. **b** Gene Ontology (GO) analysis results of differentially expressed proteins in OE33 sh-NT group and OE33 sh-GDF15 + rGDF15 group as compared to OE33 sh-GDF15 group. **c** Gene Ontology (GO) analysis results of differentially expressed proteins in OE33 sh-NT group as compared to OE33 sh-GDF15 group. Top 10 enriched pathways in biological process (BP) category, cellular component (CC) category and molecular function (MF) category are shown. Each bar represents a specific pathway, with the length of the bar corresponding to enrichment score for the enrichment. The most enriched categories in our comparison are related to mitochondrial metabolism. **d** Gene Set Enrichment Analysis (GSEA) of different expressed proteins in OE33 sh-NT and OE33 sh-GDF15 groups. Reactive oxygen species, mitochondrial gene expression and oxidative phosphorylation are significantly enriched in OE33 sh-NT group. **e** GDF15 blockade partially attenuates cisplatin-induced mitochondrial hyperpolarization in DAPI-negative OE33 cells. Representative flow cytometry histograms showing TMRE fluorescence intensity in DAPI-negative singlet cells following treatment with control medium, GDF15-neutralizing antibody (10 μg/mL), IgG1 isotype control antibody (10 μg/mL), cisplatin (3 μM), or cisplatin (3 μM) plus GDF15-neutralizing antibody (10 μg/mL). TMRE fluorescence is displayed on a log scale. Quantification of TMRE median fluorescence intensity in DAPI-negative singlet cells, normalized to the mean value of the control group. Cisplatin (3 μM) markedly increased TMRE signal in the DAPI-negative cell population, whereas GDF15 blockade partially reduced cisplatin-induced TMRE elevation. Data are presented as mean ± SD, *n* = 3. Statistical significance was determined by ordinary one-way ANOVA followed by Tukey’s multiple comparisons test. ns: *p* > 0.05, **p < 0.01. **f**-**g** Seahorse XF cell mitochondrial stress test showed the real-time oxygen consumption rate (OCR) of OE33/OE19, OE33/OE19 after co-culture with TBE63 for seven days, and the co-culture groups with consistently 1 µM AKT inhibitor VIII (AKTi) supplement. Cells were sequentially treated with 2.5 µM oligomycin, 1 µM carbonyl cyanide-p-trifluoromethoxy phenylhydrazone (FCCP), and a combination of 0.5 µM rotenone and 0.5 µM antimycin A. Data are represented as mean ± SD, *n* = 6. Unpaired t test, *p < 0.05, **p < 0.01, ***p < 0.001, ****p < 0.0001. **h** Seahorse XF cell mitochondrial stress test showed the real-time OCR of OE33 sh-NT/OE33 sh-GDF15 groups and OE19 sh-NT/OE19 sh-GDF15 groups with and without 3 µM/30 µM cisplatin treatment for 24 h. Sh-GDF15 groups showed significantly decreased OCR than sh-NT groups both in basal conditions and under cisplatin treatment-induced stress. The data were presented as mean ± SD, *n* = 5 for OE33 and *n* = 4 for OE19

A total of 865 significant differentially expressed proteins were identified in the OE33 sh-GDF15 group as compared to control, where 46 proteins showed a fold change greater than two. Additionally, treatment with rGDF15 significantly enriched 459 proteins in OE33 sh-GDF15 cells, of which 31 proteins exhibited a fold change greater than two (Fig. 5a). Among the differentially regulated proteins, several mitochondria-associated factors, including the mitochondrial respiratory chain subunit NDUFB5, cytochrome c oxidase assembly factor COA3, mitochondrial histidyl-tRNA synthetase HARS2, and the mitochondrial metabolic enzyme ALDH6A1, were markedly altered following GDF15 knockdown and restored upon rGDF15 supplementation.

The Gene Ontology (GO) analysis of differentially enriched proteins in the OE33 sh-NT group and OE33 sh-GDF15 + rGDF15 group as compared to the OE33 sh-GDF15 group revealed a strong association with mitochondrial metabolism, including mitochondrial respiratory chain complex, mitochondrial electron transport, and mitochondrial translation (Fig. 5b). Consistently, the enrichment of proteins such as NDUFB5 and COA3 highlighted the involvement of oxidative phosphorylation machinery, whereas HARS2 enrichment pointed to enhanced mitochondrial translation capacity.

Further investigation into the OE33 sh-NT and OE33 sh-GDF15 groups showed significant enrichment of biological processes related to mitochondrial gene expression and translation, and cellular components such as mitochondrial ribosomes, inner membrane, matrix, protein complexes, as well as respiratory chain complexes, along with molecular functions such as oxidoreductase activity and NADH dehydrogenase activity (Fig. 5c). The differential expression of ALDH6A1 further supported a role for GDF15 in regulating mitochondrial metabolic homeostasis.

Gene Set Enrichment Analysis (GSEA) significantly enriched mitochondrial metabolism-related pathways in the OE33 sh-NT group, including reactive oxygen species, mitochondrial gene expression, and oxidative phosphorylation (Fig. 5d). Furthermore, we utilized the TCGA dataset for external validation and conducted GSEA analysis on the high and low GDF15 expression groups in EAC patients shown in Fig. 1e. Consistent with the proteomics analysis, the GDF15-high group exhibited positive enrichment of mitochondrial oxidative phosphorylation, mitochondrial electron transport, mitochondrial genome maintenance, and mitophagy-related pathways (Figure S6c).

Based on the proteomic and transcriptomic evidence linking GDF15 to mitochondrial function, we next assessed mitochondrial membrane potential by TMRE staining followed by flow cytometry. TMRE fluorescence analysis showed that GDF15 neutralization significantly decreased the mitochondrial membrane potential profile of OE33 cells under cisplatin treatment, supporting the involvement of GDF15-associated signaling in mitochondrial adaptation during chemotherapy stress (Fig. 5e).

We then measured the real-time oxygen consumption rates (OCRs) in EAC cells. Following a seven-day co-culture with EAC CAFs, both OE33 and OE19 exhibited notable improvements in mitochondrial function, showing significant enhancements in basal respiration, maximal respiration, mitochondrial ATP production, and spare respiratory capacity (Fig. 5f-g).

We next monitored the real-time OCRs in OE33 and OE19 sh-NT and sh-GDF15 cells with and without cisplatin treatment. The results revealed a significant reduction in basal respiration in the sh-GDF15 groups compared to the sh-NT groups, indicating impaired mitochondrial function (Fig. 5h, Figure S6d). Additionally, the sh-GDF15 groups exhibited decreased maximal respiration, mitochondrial ATP production, and spare respiratory capacity, suggesting compromised mitochondrial energy production. These observations were consistent after exposing cells to cisplatin treatment for 24 h, further confirming the detrimental effect of GDF15 depletion on mitochondrial dysfunction under treatment-induced stress (Fig. 5h, Figure S6d).

Finally, to determine whether mitochondrial oxidative phosphorylation contributes functionally to chemotherapy tolerance, we performed a dose titration of oligomycin A in OE33 cells. We selected a dose of 5 nM, which exhibited negligible cytotoxicity when administered alone (Figure S6e). Under cisplatin treatment, low-dose oligomycin A further reduced OE33 cell viability, even within the protective TBE63 co-culture environment, indicating that mitochondrial respiratory activity contributes to CAF-associated cisplatin tolerance (Figure S6f).

Collectively, these proteomic, transcriptomic, and functional data suggest that GDF15 is closely associated with mitochondrial respiratory programs in EAC cells. GDF15 depletion disrupts mitochondrial protein networks and impairs OCR-based respiratory function, while CAF co-culture enhances mitochondrial respiration and pharmacological inhibition of oxidative phosphorylation reduces chemotherapy tolerance. These findings support a model in which GDF15 contributes to mitochondrial adaptation and treatment resistance in EAC cells.

### GDF15 depletion attenuates AKT pathway activation in EAC cells

**Fig. 6:**
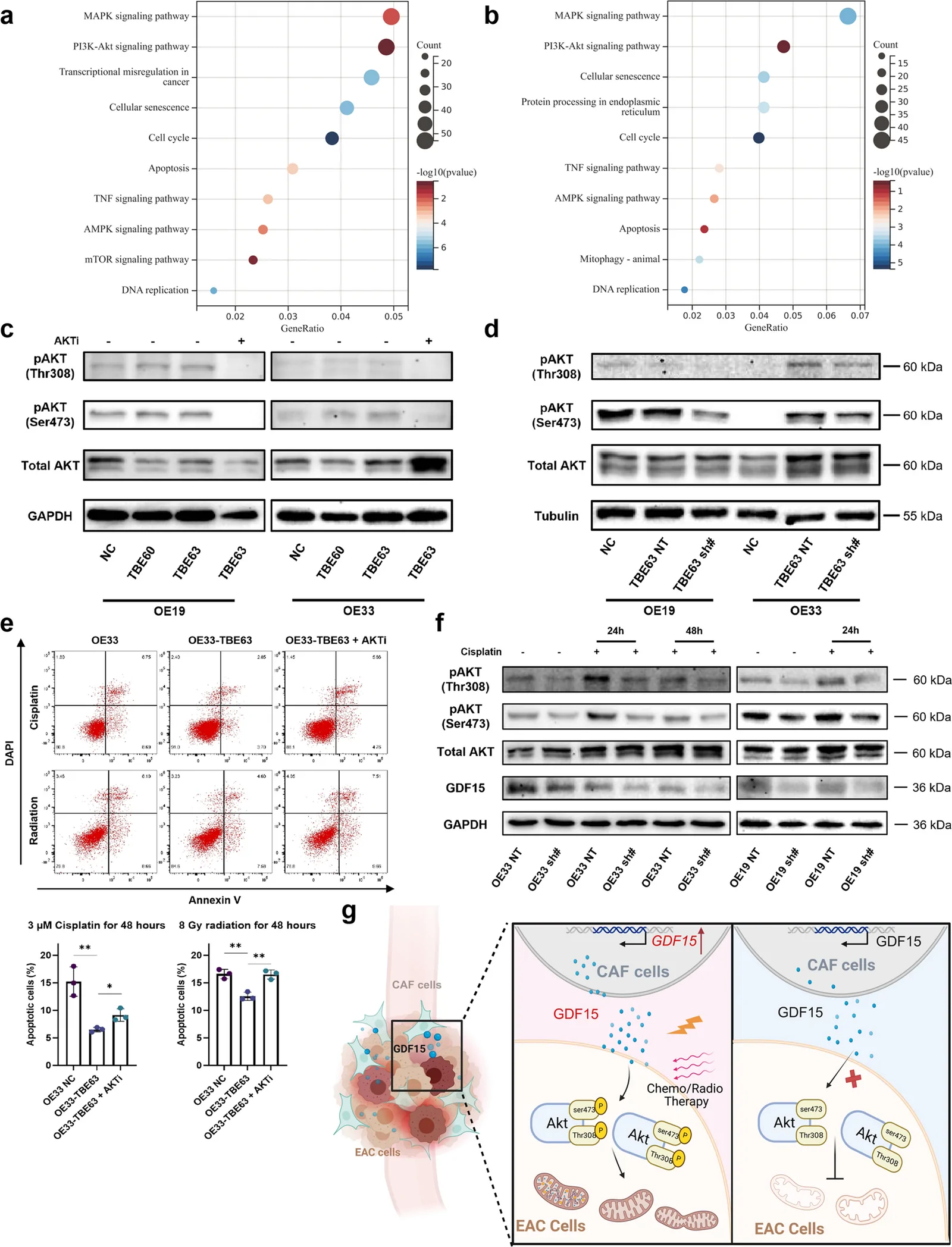
GDF15 depletion attenuates AKT pathway activation in EAC cells. **a** KEGG pathway enrichment of OE33 after co-cultured with TBE60. Each dot represents a specific pathway, and its position and size indicate the proportion and count of genes associated with a particular pathway. The color of the bar indicates the degree of significance. **b** KEGG pathway enrichment of OE33 after co-cultured with TBE63. Each dot represents a specific pathway, and its position and size indicate the proportion and count of genes associated with a particular pathway. The color of the bar indicates the degree of significance. **c** Western blot analysis shows increased levels of phosphorylated AKT (p-AKT) in OE33 and OE19 after co-culture with TBE60 and TBE63 CAFs, which can be inhibited by a consistent supplement of 1 µM AKT inhibitor VIII (AKTi). **d** Western blot analysis shows decreased levels of p-AKT in OE33 and OE19 after co-culture with TBE63 sh-GDF15 CAFs compared with TBE63 sh-NT CAFs. **e** Apoptosis assay shows that inhibiting the AKT pathway in the OE19-TBE63 co-culture system restores OE33 cell sensitivity to cisplatin and ionizing radiation, which has been enhanced by TBE63 co-culture. Data are represented as mean ± SD, *n* = 3. Unpaired t-test, *p < 0.05, **p < 0.01. **f** Western blot analysis shows GDF15 depletion results in reduced p-AKT levels in OE33 and OE19 cells, and this effect is further enhanced upon cisplatin treatment. **g** Model of the CAF-GDF15-AKT axis in mitochondrial adaptation and chemoradiation resistance in EAC

We conducted KEGG pathway enrichment analysis using the transcriptomic data previously described (Fig. 2f) and found several pathways enriched in OE33 cells following the co-culture of both TBE60 and TBE63 CAFs, including MAPK signaling pathway, PI3K-AKT signaling pathway, cellular senescence, cell cycle, apoptosis, and TNF signaling pathway (Fig. 6a-b).

AKT activation has been recognized as a key mechanism in mediating treatment resistance [40, 41]. We assessed the activation status of the AKT pathway by analyzing the protein expression levels of phosphorylated AKT (p-AKT, Thr308 and Ser473) and total AKT. The results showed increased levels of p-AKT in both OE19 and OE33 cells after co-culture with TBE60 and TBE63, indicating the activation of the AKT pathway mediated by CAFs (Fig. 6c). Besides, we observed decreased p-AKT levels when OE19 and OE33 cells were co-cultured with GDF15-depleted TBE63 CAFs, suggesting that GDF15 played a partial role in CAF-mediated AKT activation (Fig. 6d). We further investigated the impact of AKT pathway inhibition using a consistent supplement of 1 µM AKT inhibitor VIII (AKTi) in our co-culture setup. The successful inhibition of the AKT pathway in both OE19 and OE33 cells was achieved through AKTi treatment in the presence of TBE63 co-culture (Fig. 6c). Inhibiting the AKT pathway sensitized OE19 and OE33 to cisplatin and ionizing radiation treatments, indicating that CAFs enhanced EAC cell treatment resistance partially through the activation of the AKT pathway (Fig. 6e, Figure S7a). Notably, deactivation of the AKT pathway resulted in a reduction in the mitochondrial function that had been enhanced by co-culturing with TBE63, suggesting that CAFs enhanced EAC cell mitochondrial functions in part via AKT pathway activation (Fig. 5f-g). Moreover, GDF15 depletion in both OE33 and OE19 cells resulted in reduced AKT pathway activation, which was further accentuated upon treatment with cisplatin, suggesting the contribution of GDF15 to the activation of the AKT pathway in EAC cells with and without the presence of treatment stress (Fig. 6f).Consistent with this, single-cell RNA-seq analysis showed that GFRAL, the canonical receptor of GDF15, was largely low or undetectable across EAC/AEG cell populations(Figure S7b). This suggests that GDF15-mediated AKT activation may occur through GFRAL-independent or indirect mechanisms.

Taken together, these findings suggest that CAF-derived GDF15 contributes to treatment resistance in EAC cells by promoting mitochondrial function and activating the AKT signaling pathway. A schematic overview summarizing the proposed mechanism is illustrated in Fig. 6g.

### GDF15 neutralization partially attenuates CAF-associated cisplatin resistance in OE33 cells

**Fig. 7:**
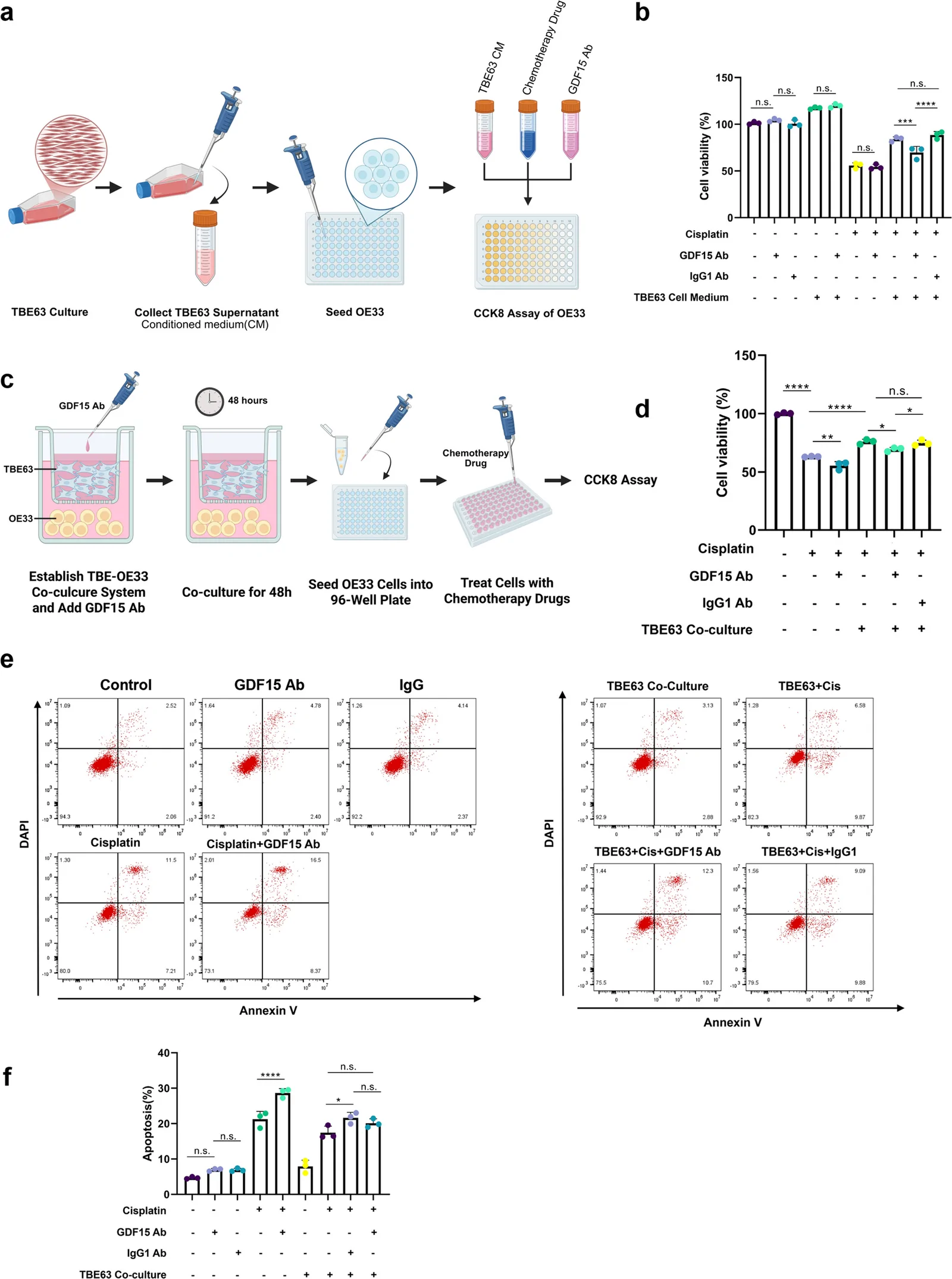
Therapeutic potential of GDF15 blockade in CAF-mediated EAC chemoresistance. **a** Schematic workflow and treatment conditions of the TBE63 CAF-conditioned medium assay. TBE63 CAF supernatant was collected after 24 h of culture. OE33 cells were then treated with the harvested conditioned medium for 48 h under cisplatin stimulation (3 μM), with or without a GDF15-neutralizing antibody (10 μg/mL) or an IgG1 isotype control (10 μg/mL). Cell viability was evaluated via CCK-8 assay. **b** Quantification of OE33 cell viability following treatment with TBE63-conditioned medium, cisplatin, and GDF15-neutralizing or IgG1 isotype control antibodies, either alone or in combination. Data are represented as mean ± SD, *n* = 3. Statistical significance was determined by ordinary one-way ANOVA followed by Tukey’s multiple comparisons test. ns: *p* > 0.05, **p < 0.01, ***p < 0.001, ****p < 0.0001. **c** Schematic workflow and treatment conditions of the TBE63-OE33 transwell co-culture assay. Following co-culture establishment, cells were treated with a GDF15-neutralizing antibody (10 μg/mL) or an IgG1 isotype control (10 μg/mL) for 48 h. Subsequently, OE33 cells were harvested and subjected to cisplatin stimulation (3 μM) for an additional 48 h. Cell viability was evaluated via CCK-8 assay. **d** Quantification of OE33 cell viability following transwell co-culture and pre-treatment with GDF15-neutralizing or IgG1 isotype control antibodies (10 μg/mL), followed by subsequent cisplatin stimulation (3 μM). Data are represented as mean ± SD, n = 3. Statistical significance was determined by ordinary one-way ANOVA followed by Tukey’s multiple comparisons test. ns: *p* > 0.05, *p < 0.05, **p < 0.01, ****p < 0.0001. **e** Representative flow cytometry plots of Annexin V-APC and DAPI staining in OE33 cells. Cells were analyzed following treatment with either control medium or TBE63 transwell co-culture, under cisplatin stimulation (3 μM) combined with or without a GDF15-neutralizing antibody (10 μg/mL) or an IgG1 isotype control (10 μg/mL). Early (Annexin V +/DAPI-) and late (Annexin V +/DAPI +) apoptotic cells were defined as indicated. **f** Quantification of total apoptotic cells, calculated as the sum of early and late apoptotic populations. Data are represented as mean ± SD, *n* = 3. Statistical significance was determined by ordinary one-way ANOVA followed by Tukey’s multiple comparisons test. ns: *p* > 0.05, *p < 0.05, ****p < 0.0001

To evaluate the translational relevance of targeting GDF15, we performed antibody-mediated GDF15-neutralization in both TBE63-conditioned medium (Fig. 7a) and transwell co-culture (Fig. 7c) systems.

Under cisplatin treatment, co-administration of the GDF15-neutralizing antibody, but not the lgG1 isotype control, partially reduced the protective effect conferred by TBE63-conditioned medium (Fig. 7b). Consistently, this rescue effect was successfully curtailed upon GDF15 blockade within the transwell co-culture setting (Fig. 7d). Furthermore, flow cytometry analysis revealed that GDF15 neutralization drove a distinct shift toward increased total apoptosis under cisplatin exposure compared with the corresponding IgG1 control (Fig. 7e-f).

Together, these findings demonstrate that GDF15 contributes to CAF-associated cisplatin resistance in EAC cells, while the partial reversal observed upon GDF15 neutralization indicates that a broader network of CAF-derived factors cooperates to support tumor cell survival.

## Discussion

In this study, we corroborate the existing literature indicating that EAC CAFs promote tumor growth and treatment resistance in EAC cells. Due to concerns that subcutaneous mouse models may not fully capture complex tumor-stroma interactions involving human CAFs [42, 43], we used tumor PDOs as a patient-derived and cost-effective model for studying EAC. The successful utilization of EAC PDOs with paired EAC CAFs for in vitro co-culture provides a valuable approach for investigating the CAF-related TME in EAC using patient-derived material. A recent study established a colorectal cancer organoid-stroma biobank containing 30 matched PDOs with CAFs and found that organoid-stroma co-cultures improved the prediction of therapy response, highlighting the potential of using organoids and paired CAFs as translational tools for personalized treatment [44]. Several studies have used different indirect and direct co-culture techniques to explore the interaction between CAFs and PDOs [45, 46]. In our study, we established both dome-based and transwell-based co-culture systems for EAC PDOs with paired CAFs. Both systems were feasible for detecting CAF-mediated effects on EAC PDO growth, while the dome co-culture system also allowed drug sensitivity assessment. To the best of our knowledge, this is the first study to use matched PDOs and CAFs to investigate the EAC tumor microenvironment. With the continued expansion of our EAC PDO and paired CAF biobank, these models may provide a useful platform for future translational and personalized therapy studies.

Limited research has focused on the role of CAF-derived GDF15 in cancer progression. Zhai et al. found that knocking down GDF15 in CAFs reduced CAF-mediated resistance to cytarabine treatment in leukemia cells [47]. Bruzzese et al. observed that prostate cancer cells exhibited enhanced proliferation, migration, and invasion when cultured with conditioned medium from GDF15-overexpressing fibroblasts [48]. In PDAC mouse models, GDF15 produced by ageing CAFs was essential for orthotopic tumor growth [49]. In our study, compartment-specific RNA-seq and ELISA analyses support that GDF15 is induced in the CAF compartment and secreted into the tumor microenvironment during EAC cell-CAF interaction. In particular, GDF15 was upregulated in TBE63 CAFs upon co-culture with OE33 cells, whereas no comparable induction was observed in the OE33 tumor-cell compartment. These findings suggest that, in our co-culture system, CAFs represent a major source of inducible GDF15. This observation is consistent with cytokine-array profiling by Ebbing et al., who reported increased GDF15 levels in EAC CAF-derived conditioned medium [50]. Nevertheless, GDF15 should be viewed as a functionally important component of a broader CAF-secreted resistance network rather than its sole mediator. Given that CAFs secrete a complex milieu of cytokines, growth factors, and metabolic regulators [50, 51], GDF15 likely acts in concert with other stromal factors to drive therapy resistance in EAC.

We found that CAFs promote EAC treatment resistance partly through GDF15 secretion. GDF15 depletion in EAC tumor cells restored sensitivity to chemotherapy and radiotherapy in vitro, indicating that GDF15 may function in treatment resistance through both CAF-derived paracrine signaling and tumor cell-intrinsic regulation. One indication of GDF15 involvement in EAC treatment resistance was observed by Karakasheva et al., who used EAC PDOs to model patient response to chemotherapy and noted common upregulation of GDF15 expression in three EAC PDOs after exposure to cisplatin and paclitaxel [52]. Another study demonstrated a protective role of GDF15 against cisplatin treatment in ESCC patients [53]. Using rGDF15, that study observed enhanced cisplatin resistance in ESCC cells and identified TGF-β receptor II (TGFBR2) as a potential GDF15 receptor [53]. However, these findings should be interpreted with caution, as recombinant GDF15 protein may be contaminated with TGF-β [54]. Given that TGFBR2 is a TGF-β receptor and that several previous studies have suggested that GDF15 does not bind to TGF-β receptors [55, 56], the observed effects in ESCC cells could potentially be attributed to TGF-β rather than GDF15. Therefore, in the present study, we used rGDF15 mainly as a rescue approach in GDF15-depleted cells to validate the functional relevance of GDF15, while avoiding overreliance on rGDF15 stimulation for downstream pathway interpretation. We found that CAFs activated the AKT pathway in EAC cells partly through GDF15 secretion, and GDF15 depletion in EAC cells decreased AKT activation. This finding is consistent with previous studies reporting CAF-mediated AKT pathway activation in ESCC [57–59]. However, most studies demonstrating GDF15-mediated AKT pathway activation in ESCC relied on rGDF15 treatment [53, 60, 61]. Notably, only one CRC-focused study has reported a correlation between fibroblast-secreted GDF15 and AKT pathway activation [62]. Our data therefore provide evidence for a CAF-associated GDF15-AKT signaling mechanism in EAC, while also suggesting that additional CAF-derived factors may cooperate with GDF15 in shaping the resistant phenotype.

Accumulating evidence suggests that mitochondrial function contributes to cancer treatment resistance, with resistant tumor cells often displaying increased reliance on mitochondrial oxidative phosphorylation and respiration but reduced reliance on glycolysis [63, 64]. We found that CAFs enhanced mitochondrial function in EAC cells, at least partly through AKT pathway activation. The association between mitochondrial function and treatment resistance in EAC was previously described by Aichler et al. in a proteomic analysis of pre-treatment biopsy samples from 23 EAC patients, where pre-existing defects in mitochondrial respiratory complexes were associated with increased chemotherapy sensitivity [65]. In addition, a recent RNA-seq study using matched EC tissue samples before and after NCRT showed enhanced OXPHOS components after NCRT, while targeting mitochondrial biogenesis in EAC cells increased sensitivity to chemotherapy and ionizing radiation [66]. In line with these observations, our study showed that inhibiting OXPHOS via oligomycin A effectively sensitized EAC cells to cisplatin treatment, even within the protective CAF co-culture environment. Furthermore, our integrated proteomic, GSEA, Seahorse, TMRE, and pharmacological OXPHOS inhibition data provide multi-dimensional evidence linking GDF15 and mitochondrial adaptation in EAC cells. While CAF co-culture effectively augmented mitochondrial respiration to support tumor cell survival, GDF15 depletion, conversely, impaired key respiratory parameters, including basal respiration, maximal respiration, mitochondrial ATP production, and spare respiratory capacity. Collectively, these findings support the interpretation that mitochondrial respiration is not merely a correlate of CAF-associated resistance, but a functional contributor to chemotherapy tolerance, driven at least in part by GDF15.

Our TMRE-based analysis further suggested that mitochondrial membrane potential is dynamically altered under chemotherapy stress. Interestingly, cisplatin treatment increased TMRE fluorescence in the viable OE33 cell population. Although mitochondrial depolarization is commonly associated with late apoptosis, increased mitochondrial membrane potential can also occur during earlier stress responses before subsequent mitochondrial collapse [67]. Consistently, cisplatin treatment has been reported to increase mitochondrial membrane potential in cancer cells and to induce mitochondrial hyperpolarization in other cisplatin-stressed cell models [68, 69]. Since dead cells were excluded before TMRE quantification, the observed increase in TMRE fluorescence likely reflects mitochondrial hyperpolarization or compensatory mitochondrial adaptation in the surviving fraction of cisplatin-treated cells, rather than the mitochondrial status of the entire treated population. GDF15 neutralization attenuated this TMRE signal under cisplatin treatment, supporting a role for GDF15-associated signaling in mitochondrial adaptation during chemotherapy-induced stress. Nevertheless, further time-course analyses will be required to distinguish transient stress-induced mitochondrial hyperpolarization from later mitochondrial depolarization during cisplatin-induced cell death.

In recent years, GDF15 has received increasing attention as an important mitokine in response to mitochondrial stress [70, 71]. Mitochondrial DNA replication defects can induce production of fibroblast growth factor 21 and GDF15 [72], and mitochondrial damage has been shown to increase GDF15 expression and protein release in normal dermal fibroblasts [73]. In this study, we did not directly investigate the mitokine role of GDF15; instead, we observed a positive regulatory association between GDF15 and mitochondrial function in EAC cells. Our findings align with previous reports showing that GDF15 depletion in human dermal fibroblasts led to accumulation of dysfunctional mitochondria [74]. CAF-derived GDF15 has also been reported to induce oxidative stress in head and neck squamous cell carcinoma [75], whereas GDF15 overexpression prevented hepatic steatosis by suppressing oxidative stress and mitochondrial damage [76]. To date, no study has reported the association between GDF15 and mitochondrial function in EAC. Future investigations should further define whether GDF15 acts primarily as a mitokine, a paracrine CAF-derived survival factor, or a broader mitochondrial regulator in the context of the CAF-related EAC microenvironment.

Corroborating previous studies [19, 20], we observed the clinical significance of GDF15 as a biomarker in EAC patients. Although several studies have reported a prognostic role of GDF15 in ESCC patients [77, 78], our data suggest a particularly relevant prognostic value of GDF15 in EAC. Unlike previous studies that measured serum GDF15 concentration only at diagnosis, we analyzed paired serum samples before and after CROSS treatment. GDF15 is widely recognized as a stress-response cytokine, with changes in both expression and serum levels observed in response to different stimuli across various disease processes [79, 80]. Elevated serum GDF15 after CROSS treatment may therefore represent a systemic stress-response signal induced by chemoradiotherapy, which may also cause emesis, anorexia and weight loss in response to platinum-containing chemotherapies [81]. Our data now showed that elevated serum GDF15 after CROSS treatment, but not at diagnosis, independently predicted poor prognosis in EAC patients. These findings highlight the potential importance of monitoring dynamic changes in serum GDF15 levels during clinical intervention in EAC. Future studies with larger sample sizes are warranted to validate the prognostic value of GDF15 and to determine how serum GDF15 dynamics could be integrated into individualized treatment strategies and precision medicine.

Several limitations should be acknowledged. First, although we incorporated additional CAF-state marker profiling, the present study did not fully resolve CAF heterogeneity in EAC. Growing evidence supports the existence of distinct CAF subtypes with both tumor-promoting and tumor-suppressing functions [51, 82]. We observed heterogeneity among the EAC CAFs used in this study, as evidenced by distinct expression profiles of fibroblast-related and CAF-state-associated markers. Despite this heterogeneity, different CAF lines consistently promoted EAC cell proliferation and treatment resistance, and these findings were further supported by paired CAF-PDO co-culture models. These results suggest that CAF-mediated treatment resistance is a robust phenotype in our experimental systems. To date, no specific CAF subtyping framework has yet been established for EAC [82]. Future studies using larger CAF collections, single-cell RNA sequencing, spatial transcriptomics, and functional subtype-specific perturbation will be required to define the contribution of distinct CAF states to GDF15 induction and therapy resistance in EAC. Second, we did not perform animal experiments in this study. This decision was based on concerns that mouse stroma may influence the behavior of human CAFs in conventional subcutaneous xenograft models. Instead, we established EAC PDOs with paired CAFs to study tumor-stroma interactions in a patient-derived in vitro system. The adoption of PDOs to reduce reliance on conventional animal models aligns with the ethical principles of 3R (Replace, Reduce, Refine) in animal research [25–27]. The consistent results obtained from matched PDO-CAF co-culture models strengthen the validity of our findings. Nevertheless, the lack of in vivo validation limits the immediate translational interpretation of our findings. Future investigations utilizing more advanced platforms, such as orthotopic co-implantation of patient-derived CAFs with EAC PDOs or humanized mouse models, will be essential to validate the therapeutic relevance of targeting the GDF15 axis in vivo*.* Third, although we performed GDF15 neutralization experiments, the current data should be interpreted as initial functional evidence rather than definitive proof that GDF15 blockade is broadly effective across all EAC-CAF interactions. GDF15 neutralization attenuated the protective effect of TBE63-conditioned medium and TBE63 co-culture under cisplatin treatment; however, the magnitude of this effect varied across experimental settings and readouts. The neutralizing antibody reduced CAF-associated cisplatin tolerance in CCK8-based viability assays, whereas its impact on apoptosis in the co-culture setting was more modest. This partial and context-dependent effect suggests that CAF-derived GDF15 contributes to, but does not fully explain, the CAF-mediated resistant phenotype. In addition, the current neutralization experiments were mainly performed in the OE33-TBE63 model under cisplatin treatment. Further validation using additional EAC cell lines, distinct CAF populations, and clinically relevant treatment contexts, including oxaliplatin-based chemotherapy and radiation-associated stress, will be required to determine the generalizability and therapeutic potential of GDF15 neutralization. Finally, the precise downstream mechanism by which GDF15 regulates mitochondrial function remains incompletely defined. Although our data link GDF15 to AKT activation, mitochondrial respiration, mitochondrial membrane potential, and chemotherapy tolerance, the receptor context and downstream molecular intermediates connecting extracellular GDF15 to mitochondrial adaptation in EAC remain to be elucidated. Expression of the only confirmed GDF15 receptor GFRAL is largely confined to brainstem neurons [56]. Clinically emerging GDF15-targeting agents, including ponsegromab and visugromab, provide an important rationale for further evaluating GDF15-directed strategies [83, 84]. Future studies should examine whether pharmacological GDF15 blockade can be combined with chemotherapy, radiotherapy, AKT inhibition, or mitochondrial metabolic inhibitors to overcome CAF-associated treatment resistance in EAC.

Taken together, our study identifies CAF-derived GDF15 as a clinically relevant and functionally important component of the EAC tumor microenvironment. Rather than acting as a single isolated mediator, GDF15 appears to function as a targetable node within a broader CAF-secreted resistance program that supports AKT signaling, mitochondrial adaptation, and therapy tolerance. These findings provide a rationale for further investigation of dynamic serum GDF15 monitoring and GDF15-targeted therapeutic strategies in EAC.

## Conclusions

This study identifies GDF15 as a clinically relevant mediator associated with treatment resistance in EAC. Elevated serum GDF15 levels following CROSS treatment, but not at diagnosis, independently predicted poor overall survival, supporting its potential as a prognostic biomarker and dynamic indicator of therapy-associated stress in EAC patients. Mechanistically, our in vitro data reveal that GDF15 links tumour-stroma crosstalk to AKT-associated mitochondrial adaptation in EAC cells. Specifically, CAFs augment mitochondrial oxidative phosphorylation and treatment tolerance in part through the GDF15-mediated activation of the AKT pathway. Genetic GDF15 depletion impaired mitochondrial respiratory parameters and reduced chemoradiation resistance, while recombinant GDF15 partially restored the resistant phenotype. In addition, low-dose pharmacological inhibition of OXPHOS further sensitized EAC cells to cisplatin, including within a CAF co-culture context, indicating that enhanced mitochondrial respiration is a functional contributor to CAF-associated chemotherapy tolerance.

Utilizing patient-derived organoids co-cultured with matched CAFs, alongside genetic depletion and antibody-mediated neutralization, we provide functional in vitro evidence that targeting the CAF-GDF15 axis can attenuate CAF-associated treatment resistance. However, the partial and context-dependent effects of GDF15 neutralization indicate that GDF15 functions as a critical node within a redundant CAF-secreted resistance program rather than as its sole determinant. Collectively, our findings position GDF15 at the intersection of tumor-stroma crosstalk, AKT signaling, mitochondrial adaptation, and therapy tolerance, highlighting its prognostic and therapeutic relevance in EAC. Further validation across additional EAC-CAF models, treatment modalities, and in vivo systems will be essential to define the broader applicability of GDF15-targeted strategies.

## Supplementary Information

Supplementary Material 1.

## Data Availability

This study did not generate new unique reagents. Research materials in this study are partly accessible through the corresponding author with a completed materials transfer agreement. Requestors should ensure compliance with relevant local and German legal requirements. RNA-seq data, proteomics data, original western blot images are available from the corresponding author on reasonable request. Any additional information required to reanalyze the data reported in this paper is available from the corresponding author upon appropriate request. RNA-seq data, proteomics data, original western blot images are available from the corresponding author on reasonable request. Any additional information required to reanalyze the data reported in this paper is available from the corresponding author upon appropriate request.

## Abbreviations

2D: Two-dimensional
3D: Three-dimensional
5-FU: Fluorouracil
AGEJ: Adenocarcinoma of gastroesophageal junction
ANOVA: One-way analysis of variance
CAA: Chloroacetamide
CAFs: Cancer-associated fibroblasts
CI: Confidence interval
CRC: Colorectal carcinoma
CROSS: Dutch Chemoradiotherapy for Oesophageal Cancer followed by Surgery Study
DFS: Disease-free survival
DTT: Dithiothreitol
EAC: Esophageal adenocarcinoma
EC: Esophageal cancer
ECAR: Extracellular acidification rate
ECM: Extracellular matrix
ELISA: Enzyme-linked immunosorbent assay
ESCC: Esophageal squamous cell carcinoma
FCCP: Carbonyl cyanide-p-trifluoromethoxy phenylhydrazone
FGF21: Fibroblast growth factor 21
GDF15: Growth differentiation factor 15
GEO: Gene Expression Omnibus
GO analysis: Gene Ontology analysis
GSEA: Gene Set Enrichment Analysis
H&E: Hematoxylin-eosin
HR: Hazard ratio
HRP: Horseradish peroxidase
IF: Immunofluorescence
IHC: Immunohistochemistry
IL-6: Interleukin 6
LFQ: Label-free quantification
lnHR: Natural logarithm of the hazard ratios
Lys-C: Lysyl endopeptidase
mtDNA: Mitochondrial DNA
NCRT: Neoadjuvant chemoradiotherapy
NCT: Neoadjuvant chemotherapy
OCR: Oxygen consumption rate
OS: Overall survival
OXPHOS: Mitochondrial oxidative phosphorylation
p-AKT: Phosphorylated AKT
pCR: Pathological complete response
PD-1: Programmed death 1
PDOs: Patient-derived tumor organoids
PEI: Polyethylenimine
PFS: Progression-free survival
qPCR: Quantitative Real-time PCR
RCTs: Randomized controlled trials
rGDF15: Recombinant GDF15 protein
RNA-seq: RNA sequencing
RoB2 tool: Risk-of-bias tool
Rot/AA: Rotenone/antimycin A
SD: Standard deviation
TCGA: The Cancer Genome Atlas
TEAB: Triethylammonium bicarbonate
TGF-β1: Transforming growth factor-β1
TME: Tumor microenvironment
GDF15 Ab: GDF15-neutralizing antibody
IgG1 Ab: Invivomab Mouse IgG1 isotype control
