# Correction: Targeting the CAF-GDF15 axis attenuates AKT-mediated mitochondrial rewiring and chemoradiation resistance in esophageal adenocarcinoma

**DOI:** 10.1186/s13046-026-03859-4

**Published:** 2026-11-06

**Authors:** Ningbo Fan, Pinwei Deng, Zhefang Wang, Feng Ju, Lisa Raatz, Oscar Velazquez Camacho, Axel M. Hillmer, Margarete Odenthal, Phuong-Hien Nguyen, Zicheng Lyu, Felix Popp, Seung-Hun Chon, Maarten F. Bijlsma, Romy Ros, Hanneke W. M. van Laarhoven, Alexander Quaas, Jörg Wischhusen, Yue Zhao, Christiane J. Bruns

**Affiliations:** 1 Department of General, Visceral, Thoracic and Transplantation Surgery, University Hospital of Cologne, Cologne, 50937 Germany; 2 Institute of Pathology, University of Cologne, Faculty of Medicine and University Hospital of Cologne, Cologne, 50937 Germany; 3 Center for Molecular Medicine Cologne, University of Cologne, Cologne, 50937 Germany; 4 Department I of Internal Medicine, University of Cologne, Faculty of Medicine and University Hospital Cologne, Center for Integrated Oncology Aachen Bonn Cologne Duesseldorf, Center for Molecular Medicine Cologne, Cologne, Cologne, 50937 Germany; 5 Laboratory for Experimental Oncology and Radiobiology, Center for Experimental and Molecular Medicine, Amsterdam UMC, University of Amsterdam, RDC Adore, Amsterdam, The Netherlands; 6 Cancer Center Amsterdam, Imaging and Biomarkers, Amsterdam, the Netherlands; 7 Department of Medical Oncology, Amsterdam UMC, University of Amsterdam, Amsterdam, The Netherlands; 8 Department of Thoracic Oncology, State Key Laboratory of Oncology in South China, Collaborative Innovation Center for Cancer Medicine, Sun Yat-Sen University Cancer Center, Guangzhou, 510060 China; 9 Department for Obstetrics & Gynaecology, University Hospital Würzburg, Section for Experimental Tumor Immunology, Josef-Schneider-Str. 4, Würzburg, 97080 Germany

**Correction: J Exp Clin Cancer Res 45**,** 164 (2026)**

**https://doi.org/10.1186/s13046-026-03776-6**

Following publication of the original article [1], the authors found error in Fig. 3k. the representative flow cytometry image for Group OE19 sh# + 50ng/ml rGDF15 was mistakenly duplicated with the image from Group OE19 sh#.

The correct figure is given below and the original article [1] has been corrected.

**Current Fig. ****3****k**.

**Fig. 3:**
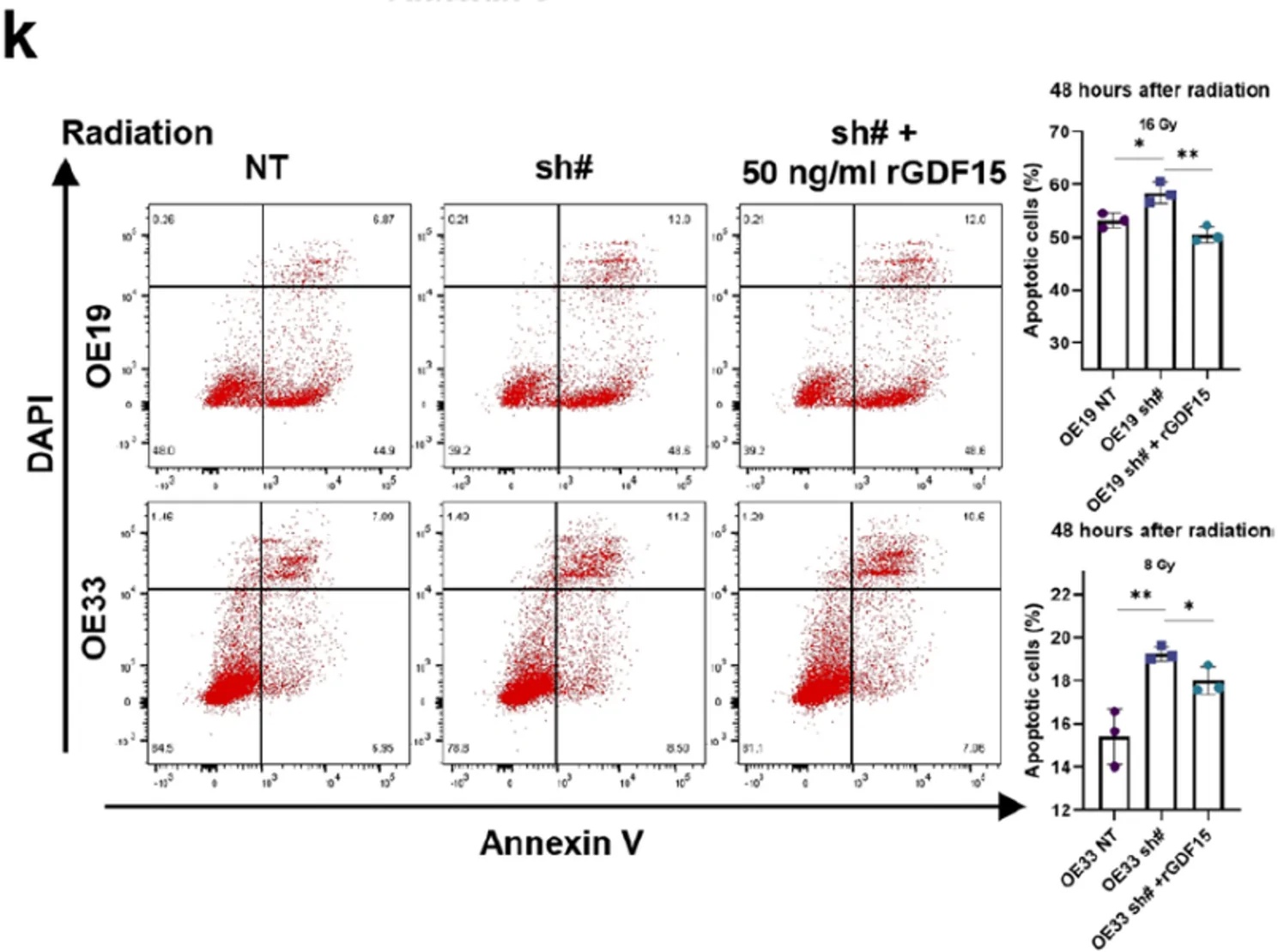
GDF15 depletion results in reduced treatment resistance in EAC cells and reduced EAC CAF-mediated treatment resistance in vitro. **a** GDF15 concentrations are significantly higher in the co-culture medium in both OE33 and OE19 cells. Data are represented as mean ± SD, *n* = 3. Unpaired t-test, **p < 0.01, ***p < 0.001, ****p < 0.0001. **b**-**c** Validation of GDF15 knockdown (sh#) and control (NT) tumor cells in qPCR and western blot. Data are represented as mean ± SD, *n* = 3. Unpaired t-test, ****p < 0.0001. **d** Validation of GDF15 knockdown and control CAF cell line TBE63 in qPCR and western blot. Unpaired t-test, *n* = 3, ****p < 0.0001. **e** Transwell co-culture setup of co-culturing EAC cell lines with GDF15-depleted/control TBE63 cells. GDF15 concentrations are significantly decreased in OE19/OE33-TBE63 GDF15 knockdown co-culture medium. Data are represented as mean ± SD, *n* = 3. Unpaired t-test, **p < 0.01, ***p < 0.001. **f** Cell viability assay shows increased sensitivity to cisplatin and oxaliplatin treatments after GDF15 knockdown in OE19 and OE33 cells. Data are represented as mean ± SD, *n* = 3. **g** Cell viability assay shows increased sensitivity to oxaliplatin and cisplatin treatment in OE19 and OE33 cells after co-culture with GDF15-depleted TBE63. Data are represented as mean ± SD, *n* = 3. **h** Apoptosis assay shows increased apoptotic populations in OE19 and OE33 GDF15 knockdown cells 48 h after exposure to ionizing radiation (16 Gy and 8 Gy, respectively). Data are represented as mean ± SD, *n* = 3. Unpaired t-test, *p < 0.05, **p < 0.01. **i** Apoptosis assay shows increased apoptotic populations in OE19 and OE33 GDF15 knockdown cells after 48 h cisplatin treatment (30 µM and 3 µM, respectively). Data are represented as mean ± SD, *n* = 3. Unpaired t-test, *p < 0.05, **p < 0.01. **j**-**k** Rescue assays show that exogenous human rGDF15 treatment restores resistance to chemotherapy and ionizing radiation in GDF15-depleted OE19 and OE33 cells. The apoptosis assay is performed to determine cell sensitivity by calculating the apoptotic cell populations after the treatment. Data are represented as mean ± SD, *n* = 3. Unpaired t-test, *p < 0.05, **p < 0.01

**Correct Fig. ****3****k**.

**Fig. 3:**
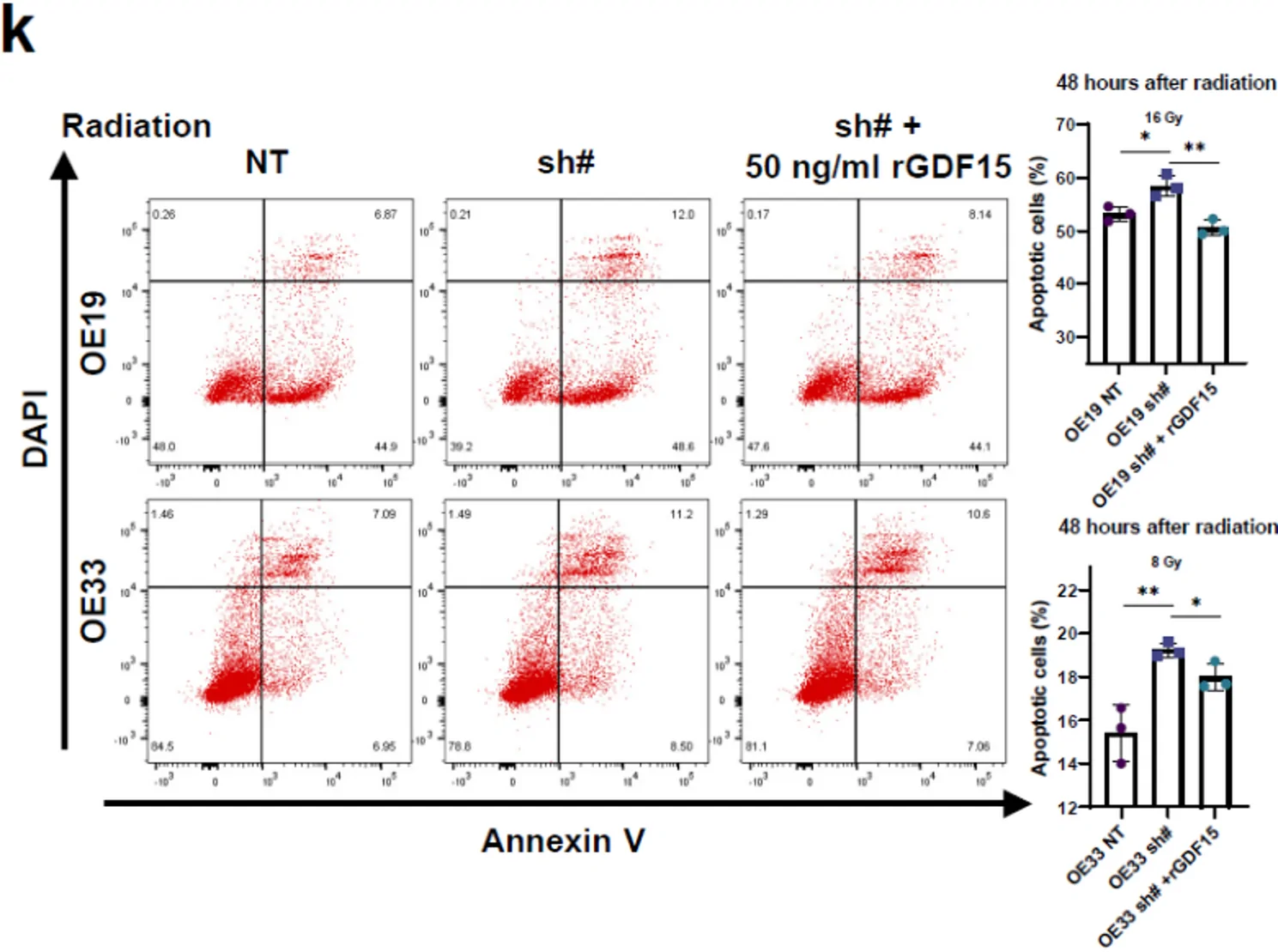
GDF15 depletion results in reduced treatment resistance in EAC cells and reduced EAC CAF-mediated treatment resistance in vitro. **a** GDF15 concentrations are significantly higher in the co-culture medium in both OE33 and OE19 cells. Data are represented as mean ± SD, n = 3. Unpaired t-test, **p < 0.01, ***p < 0.001, ****p < 0.0001. **b**-**c** Validation of GDF15 knockdown (sh#) and control (NT) tumor cells in qPCR and western blot. Data are represented as mean ± SD, n = 3. Unpaired t-test, ****p < 0.0001. **d** Validation of GDF15 knockdown and control CAF cell line TBE63 in qPCR and western blot. Unpaired t-test, n = 3, ****p < 0.0001. **e** Transwell co-culture setup of co-culturing EAC cell lines with GDF15-depleted/control TBE63 cells. GDF15 concentrations are significantly decreased in OE19/OE33-TBE63 GDF15 knockdown co-culture medium. Data are represented as mean ± SD, n = 3. Unpaired t-test, **p < 0.01, ***p < 0.001. **f** Cell viability assay shows increased sensitivity to cisplatin and oxaliplatin treatments after GDF15 knockdown in OE19 and OE33 cells. Data are represented as mean ± SD, n = 3. **g** Cell viability assay shows increased sensitivity to oxaliplatin and cisplatin treatment in OE19 and OE33 cells after co-culture with GDF15-depleted TBE63. Data are represented as mean ± SD, n = 3. **h** Apoptosis assay shows increased apoptotic populations in OE19 and OE33 GDF15 knockdown cells 48 h after exposure to ionizing radiation (16 Gy and 8 Gy, respectively). Data are represented as mean ± SD, n = 3. Unpaired t-test, *p < 0.05, **p < 0.01. **i** Apoptosis assay shows increased apoptotic populations in OE19 and OE33 GDF15 knockdown cells after 48 h cisplatin treatment (30 µM and 3 µM, respectively). Data are represented as mean ± SD, n = 3. Unpaired t-test, *p < 0.05, **p < 0.01. **j**-**k** Rescue assays show that exogenous human rGDF15 treatment restores resistance to chemotherapy and ionizing radiation in GDF15-depleted OE19 and OE33 cells. The apoptosis assay is performed to determine cell sensitivity by calculating the apoptotic cell populations after the treatment. Data are represented as mean ± SD, n = 3. Unpaired t-test, *p < 0.05, **p < 0.01
