# Supplementary material for: Targeting the CAF-GDF15 axis attenuates AKT-mediated mitochondrial rewiring and chemoradiation resistance in esophageal adenocarcinoma
Source: J Exp Clin Cancer Res. 2026 Jul 17;45:164. doi: 10.1186/s13046-026-03776-6 (PMC13378158; doi:10.1186/s13046-026-03776-6)
Supplement: Supplementary file 1 — Supplementary Material 1. [file 13046_2026_3776_MOESM1_ESM.docx]

**Supplemental information**

**(1) Supplemental figures:**

[**Supplemental figure 1.**](#FigureS1) Clinical significance of serum GDF15 concentration in EAC patients from the Amsterdam UMC.

[**Supplemental figure 2.**](#FigureS2) Validation of EAC CAFs.

[**Supplemental figure 3.**](#FigureS3) EAC CAFs promote EAC cell proliferation and treatment resistance *in vitro*.

[**Supplemental figure 4.**](#FigureS4) GDF15 depletion does not affect the proliferation of EAC cells.

[**Supplemental figure 5.**](#FigureS5) Growth kinetics of EAC PDOs with and without transwell co-cultured with paired CAFs.

[**Supplemental figure 6.**](#FigureS6) GDF15 depletion results in impaired mitochondrial function in EAC cells.

[**Supplemental figure 7.**](#FigureS7) GDF15 depletion attenuates AKT pathway activation in EAC cells.

**(2) Supplemental tables:**

[**Supplemental table 1.**](#TableS1) Characteristics of the EAC patients for EAC CAFs included in this study.

[**Supplemental table 2.**](#TableS2) Comparison of patient characteristics in serum GDF15 low group and high group before and after CROSS treatment.

[**Supplemental table 3.**](#TableS3) Characteristics of the EAC patients with paired EAC tumor and adjacent normal tissue samples.

[**Supplemental table 4.**](#TableS4) List of primers.

**(3) Key resource table**

[**Key resource table**](#KRT)

**
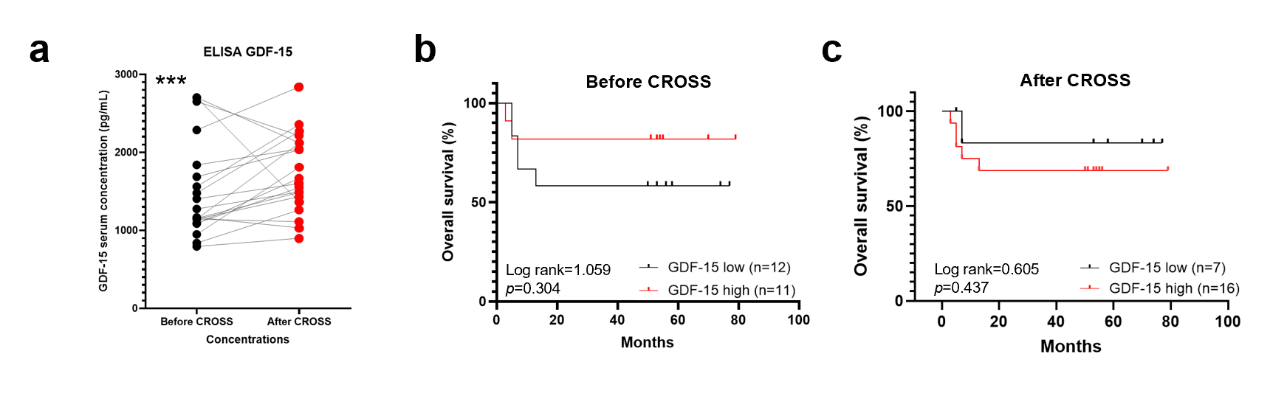
Supplemental figure 1. Clinical significance of serum GDF15 concentration in EAC patients from the Amsterdam UMC.**

1. Paired serum GDF15 concentration before and after the CROSS treatment in 23 EAC patients. Serum GDF15 is significantly higher after the CROSS treatment. Paired t-test, ****p* < 0.001.

b. Kaplan–Meier survival analysis according to serum GDF15 levels before and after CROSS treatment. No significant difference in overall survival was observed between patients with high and low serum GDF15 levels before CROSS treatment (Log-rank = 1.059, *p* = 0.304). Following CROSS treatment, patients with elevated serum GDF15 levels exhibited a trend toward poorer overall survival, although the difference did not reach statistical significance (Log-rank = 0.605, *p* = 0.437).

**CAF3095**


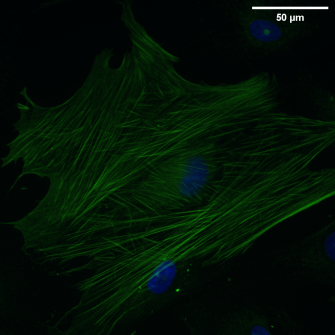

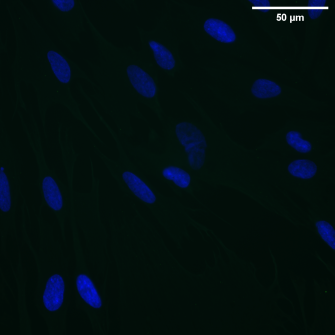


**CAF2304**


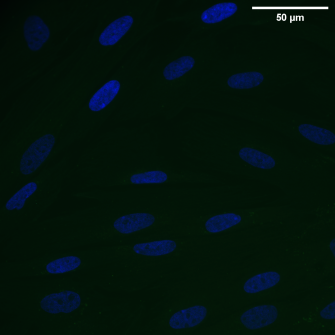

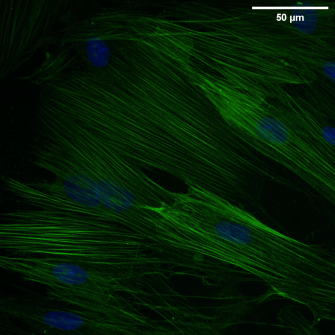


**CAF2765**

**αSMA**

**DAPI**

**Pan-CK**

**DAPI**


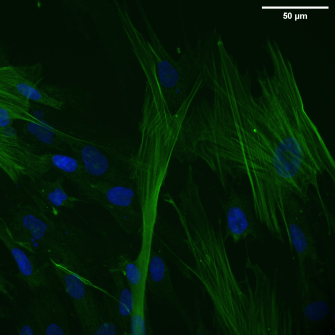

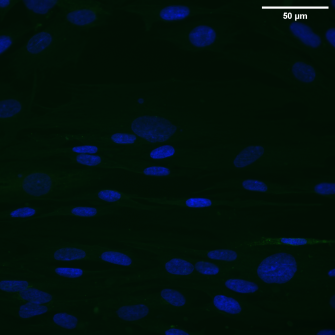


**OE33**

**αSMA**

**DAPI**

**Pan-CK**

**DAPI**


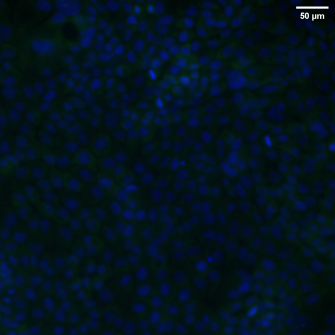

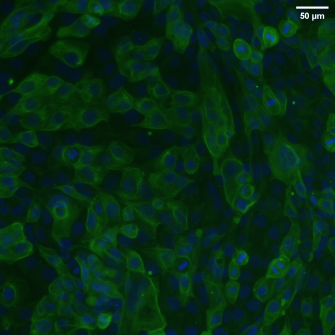

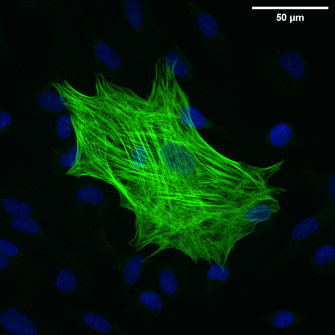


**TBE60**


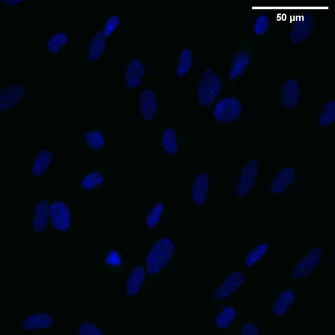

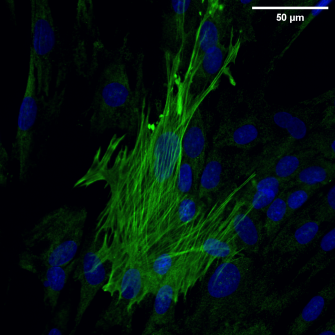

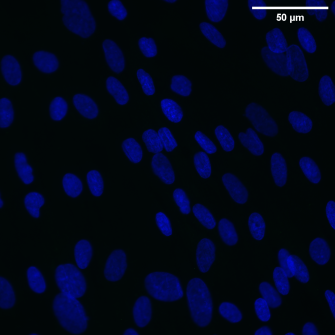


**TBE63**

**a**

**b**

**c**


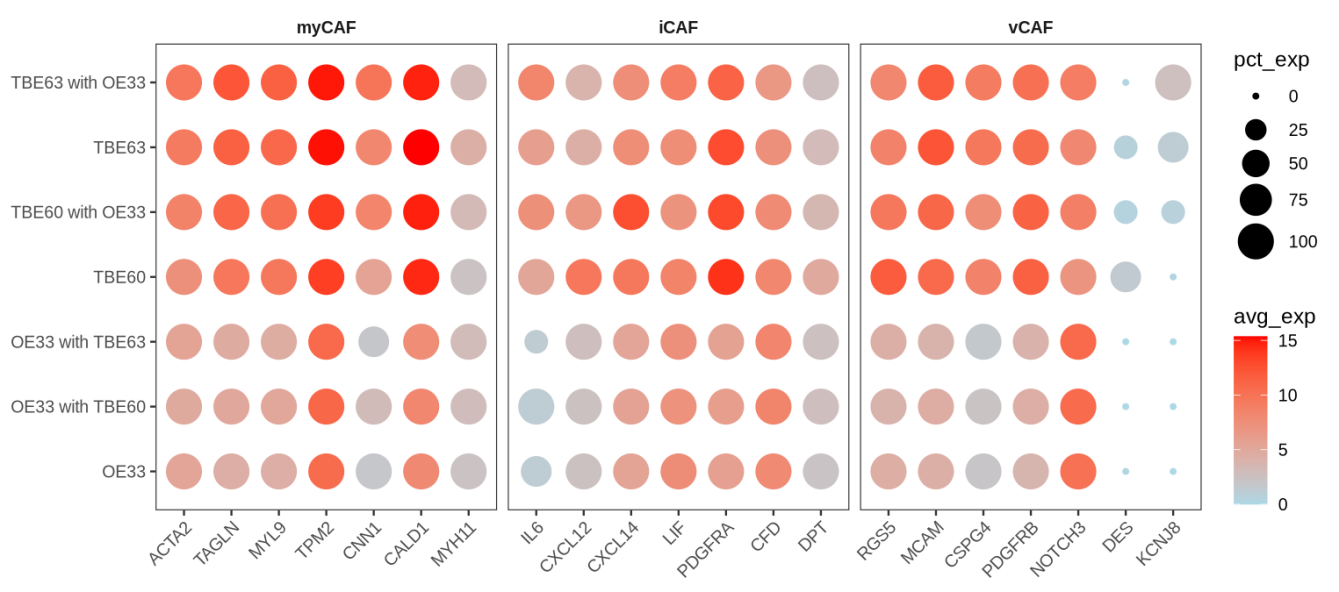


**Supplemental figure 2. Validation of EAC CAFs.**

a. The immunofluorescence (IF) staining of epithelial marker Pan-CK, fibroblast marker αSMA, and DAPI in EAC cell line OE33 and different EAC CAFs TBE60, TBE63, CAF2304, CAF2765 and CAF3095. OE33 is Pan-CK positive but αSMA negative, while all the CAFs express αSMA but not Pan- CK. Scale bar: 50µm.

b. Relative mRNA expression of fibroblast markers αSMA, IL-6, PDGFRα and Vimentin in EAC cell lines OE19 and OE33 and EAC CAFs TBE60, TBE63, CAF2304, CAF2765 and CAF3095. Data are represented as mean ± SD, n=3.

c. Dot plot showing the expression of selected CAF subtype-associated marker genes across OE33 cells, TBE60 and TBE63 CAFs, and OE33–CAF co-culture conditions. Genes were categorized into myCAF-, iCAF-, and vCAF-associated marker panels. Dot size indicates the percentage of cells expressing each gene, and dot color represents the average expression level. The plot illustrates distinct CAF marker expression profiles between OE33 tumor cells and CAF populations, as well as altered CAF-associated expression patterns under co-culture conditions.

**
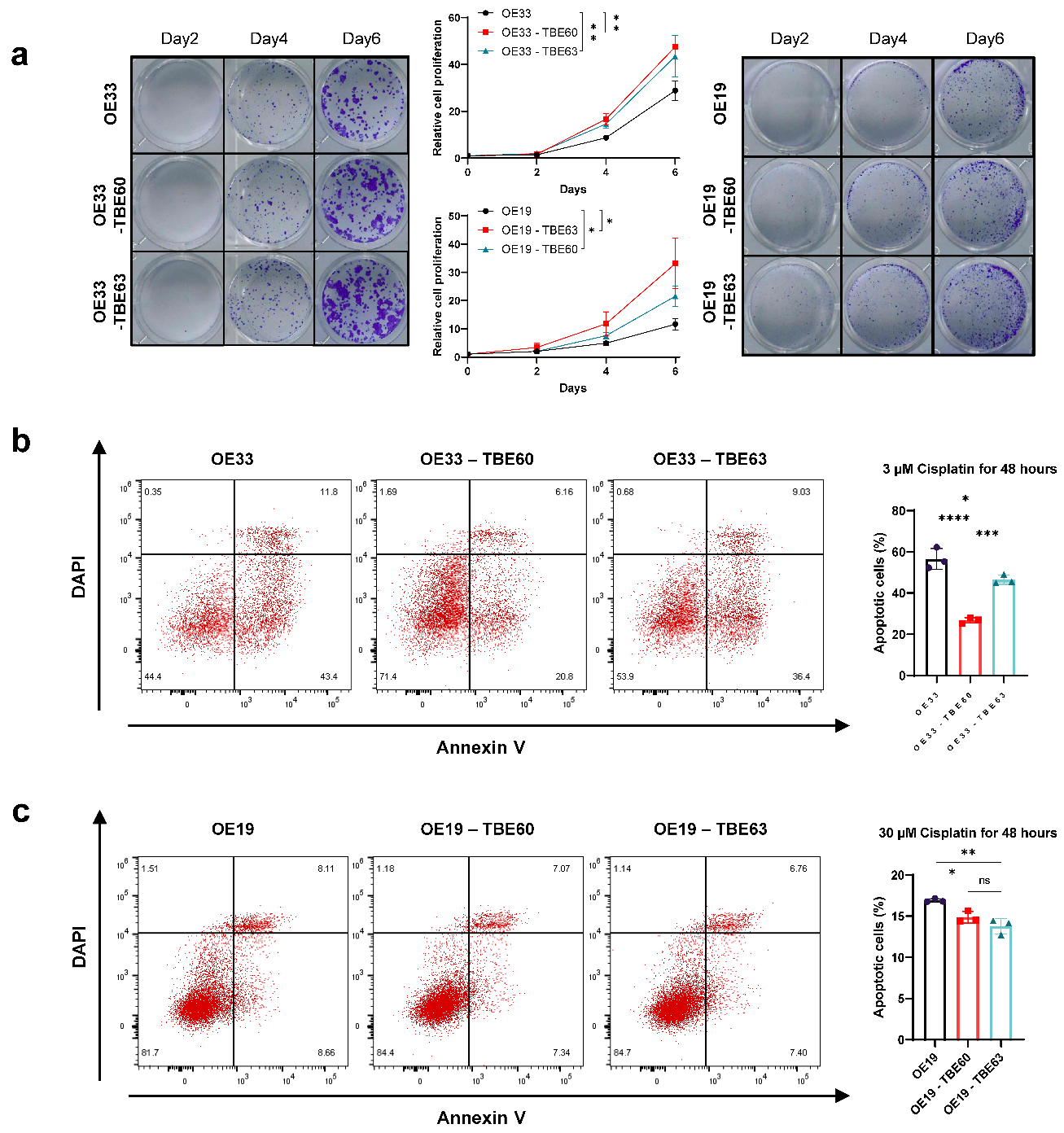
****Supplemental figure 3. EAC CAFs promote EAC cell proliferation and treatment resistance *in vitro*.**

1. Cell proliferation assay shows that both EAC CAFs TBE60 and TBE63 promote OE33 and OE19 cell proliferation *in vitro.* Data are represented as mean ± SD, n=3. Paired t test, **p* < 0.05.
2. Apoptosis assay shows that after co-culture with two different EAC CAFs TBE60 and TBE63, OE33 has fewer apoptotic populations after 48 hours of 3 µM cisplatin treatment *in vitro*. Data are represented as mean ± SD, n=3. Unpaired t-test, **p* < 0.05, ****p* < 0.001, *****p* < 0.0001.
3. Apoptosis assay shows that after co-culture with two different EAC CAFs TBE60 and TBE63, OE19 has fewer apoptotic populations after 48 hours of 30 µM cisplatin treatment *in vitro*. Data are represented as mean ± SD, n=3. Unpaired t-test, ns: *p* > 0.05, **p* < 0.05, ***p* < 0.01.

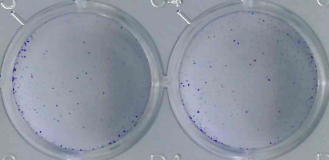

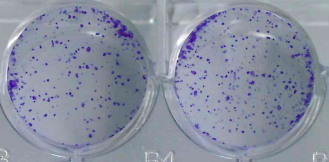

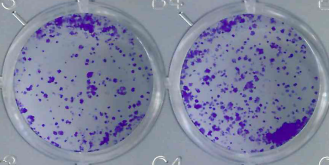


**OE33 sh#**

**OE33 NT**

Day 2

Day 4

Day 6

**a**

**OE19 NT**

**OE19 sh#**

Day 2

Day 4

Day 6


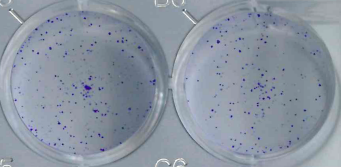

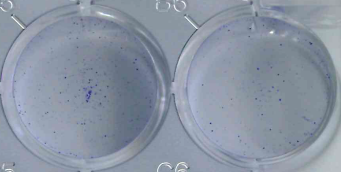

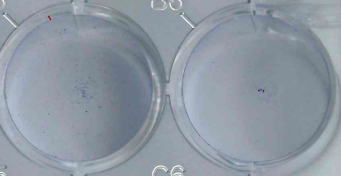


**b**

**Supplemental figure 4. GDF15 depletion does not affect the proliferation of EAC cells.**

a. Cell proliferation assay shows no proliferation difference after GDF15 knockdown in OE33. Data are represented as mean ± SD, n=3. Paired t-test, ns: *p* > 0.05.

b. Cell proliferation assay shows no proliferation difference after GDF15 knockdown in OE19. Data are represented as mean ± SD, n=3. Paired t-test, ns: *p* > 0.05.

**Supplemental figure 5. Growth kinetics of EAC PDOs with and without transwell co-cultured with paired CAFs.**

Day 0

Day 4

Day 6

Day 8

Day 10


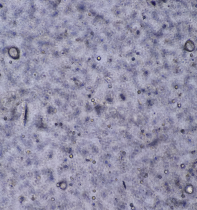

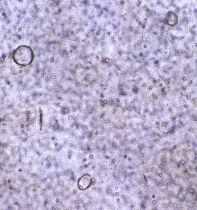

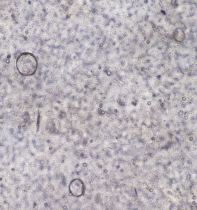

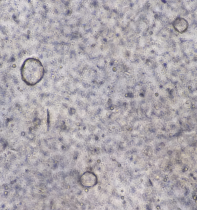

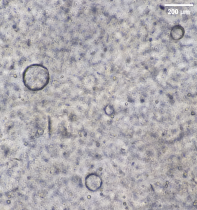


+ CAF2765


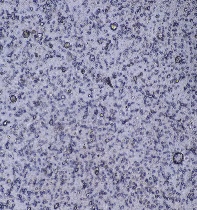

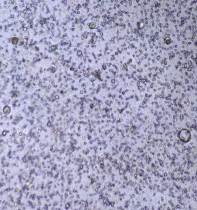

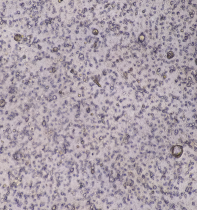

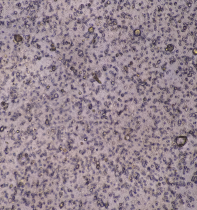

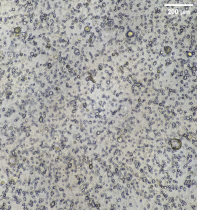


PDO2765

PDO sizes are tracked under an ECHO Rebel microscope, and the corresponding areas of PDOs are measured and digitized using ECHO pro (v6.4.1) software.

**NES:1.630**

***P value*: 0.080**

**d**

**c**

**e**

**a**


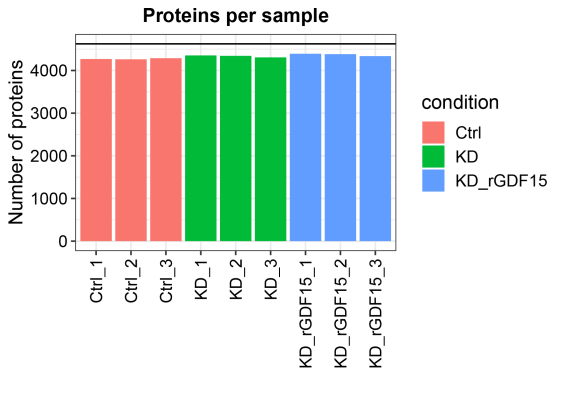

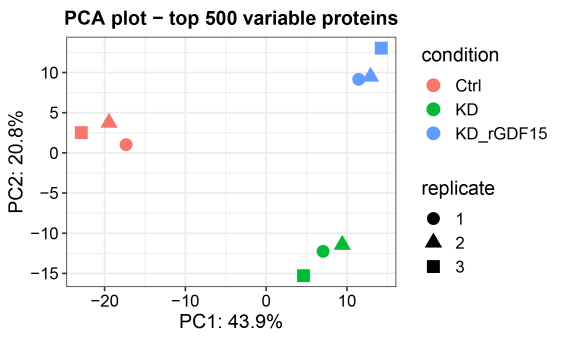


**TCGA database/EAC**

**NES:1.630**

***P value*: 0.080**


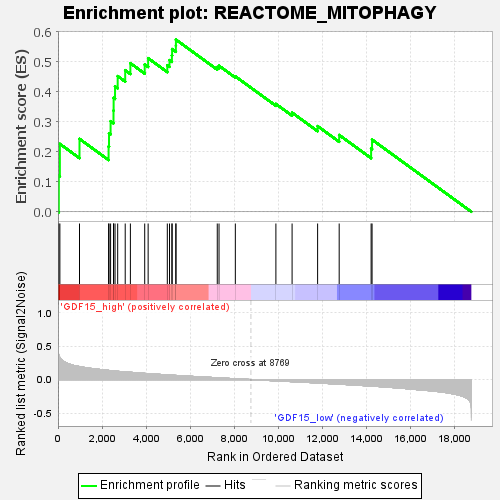

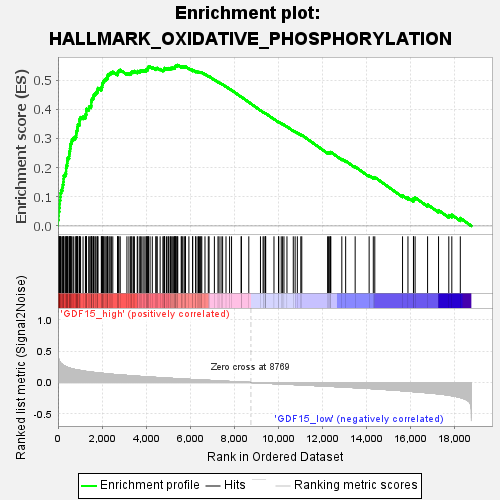

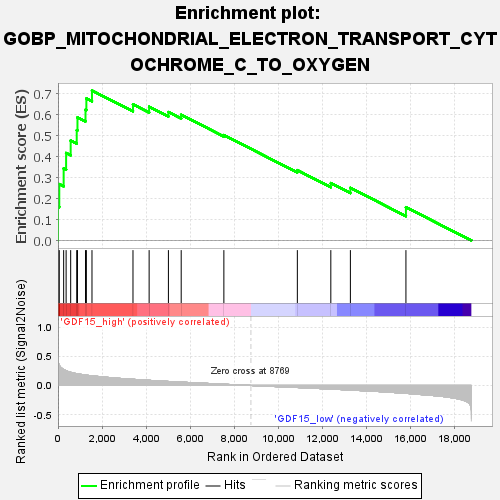

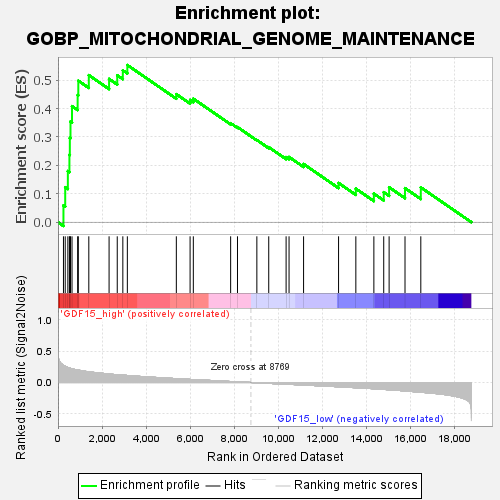


**NES:1.668**

***P value*: 0.031**

**NES:1.727**

***P value*: 0.018**

**NES:1.753**

***P value*: 0.012**


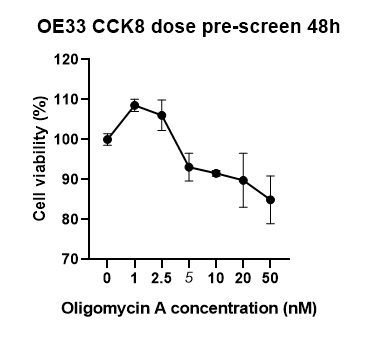


**b**

**f**


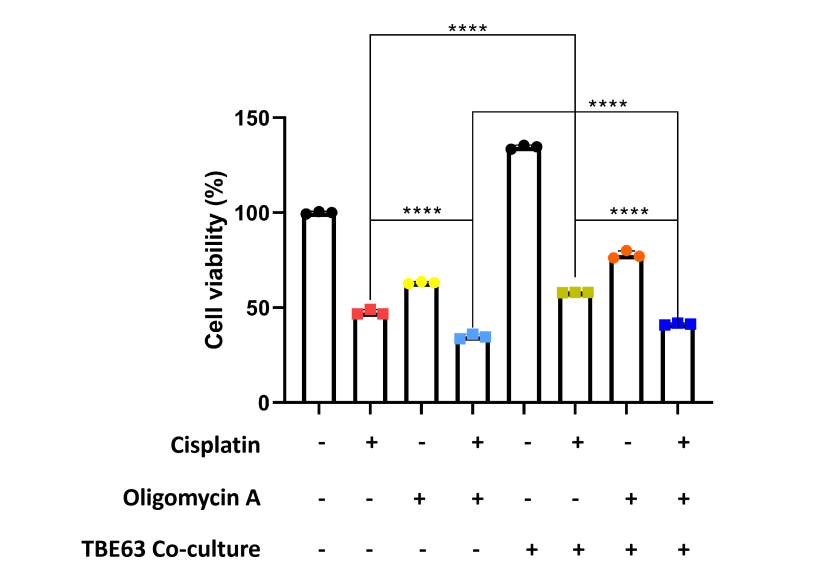


**Supplemental figure 6. GDF15 depletion results in impaired mitochondrial function in EAC cells.**

a-b. The quality control of protein omics data. Numbers of proteins of each group with three biological replicants are shown on a, and principal component analysis (PCA) is shown on b. More than 4000 proteins are identified in each sample and distinct protein expression profiles are achieved among the experimental groups.

c. Gene Set Enrichment Analysis (GSEA) of differently expressed proteins in GDF15 high expression group and GDF15 low expression group in EAC patients from the TCGA database. The y-axis represents the enrichment score, and the x-axis represents the rank of the genes. The NES and the corresponding *p*-value are indicated. Mitochondrial function and mitochondrial homeostasis related pathways are enriched in GDF15 high expression group.

d. Seahorse XF cell mitochondrial stress test shows the real time oxygen consumption rate (OCR) of OE33 sh-NT and OE33 sh-GDF15 groups with and without 3 µM cisplatin treatment for 24 hours, and OE19 sh-NT and OE19 sh-GDF15 groups with and without 30 µM cisplatin treatment for 24 hours. Statistical analysis of Figure 5h. Data are represented as mean ± SD, n=5 for OE33 and n=4 for OE19. Unpaired t-test, **p* < 0.05, ****p* < 0.001.

e. Dose–response analysis of oligomycin A in OE33 cells. OE33 cells were treated with increasing concentrations of oligomycin A for 48 hours, and cell viability was normalized to the untreated control group. A low concentration of oligomycin A was selected for subsequent combination treatment to minimize its single-agent cytotoxicity. Data are represented as mean ± SD, n=3.

f. CCK8 assay of OE33 cells cultured alone or co-cultured with TBE63 CAFs following treatment with cisplatin (3 μM), oligomycin A (5 nM), or both. Low-dose oligomycin A enhanced cisplatin-induced growth inhibition in both monoculture and TBE63 co-culture conditions. Data are represented as mean ± SD, n=3. Ordinary one-way ANOVA with Tukey’s multiple comparisons test, *****p* < 0.0001.


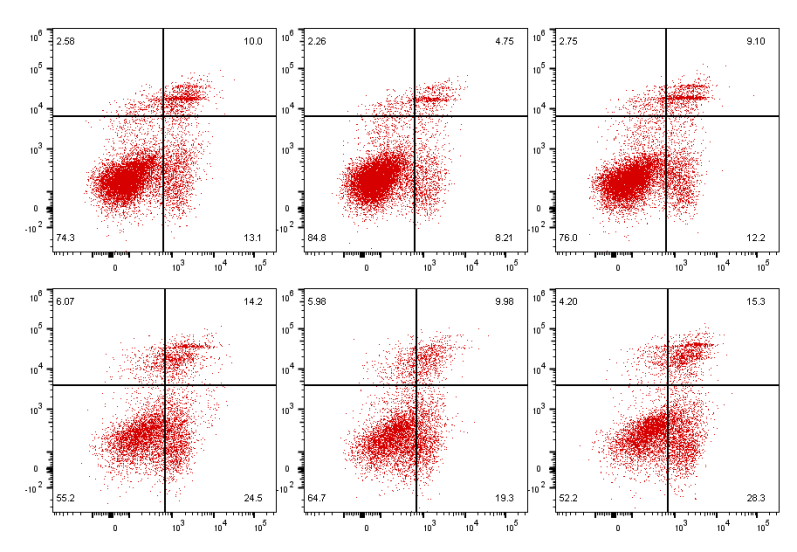


**Annexin V**

**DAPI**

**Radiation**

**OE19-TBE63**

**OE19**

**OE19-TBE63 + AKTi**

**Cisplatin**

**a**

**b**


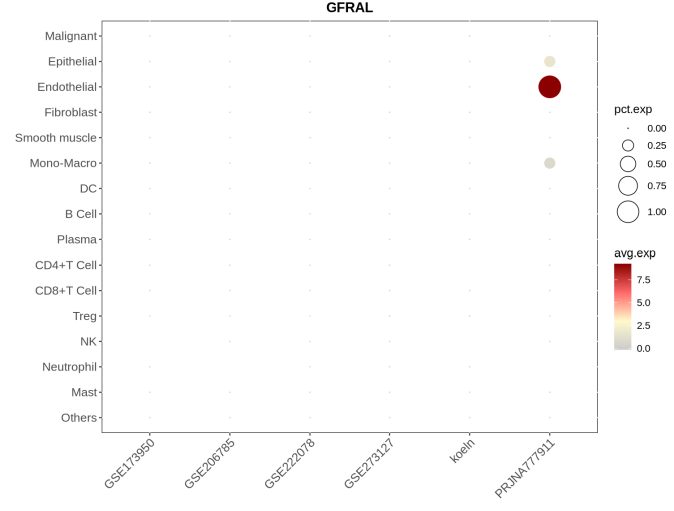


**Supplemental figure 7. GDF15 depletion attenuates AKT pathway activation in EAC cells.**

a. Apoptosis assay shows that inhibiting the AKT pathway in the OE19-TBE63 co-culture system restores OE19 cell sensitivity to cisplatin and ionizing radiation, which has been enhanced by TBE63 co-culture. The apoptosis assay is performed to determine cell sensitivity by calculating the apoptotic cell populations after the treatment. Data are represented as mean ± SD, n=3. Unpaired t-test, **p* < 0.05, ***p* < 0.01.

b. Dot plot showing GFRAL mRNA expression across major annotated cell populations in five public single-cell RNA-sequencing cohorts (GSE173950, GSE206785, GSE222078, GSE273127, and PRJNA777911) and one in-house cohort. Dot size represents the percentage of cells expressing GFRAL within each cell population (pct.exp), while dot color indicates the average expression level (avg.exp). All analyzed samples were derived from esophageal adenocarcinoma (EAC) or adenocarcinoma of the esophagogastric junction (AEG/AEJ). For mixed cohorts, only EAC/AEJ samples were retained: EAC samples from GSE273127, AEJ samples from GSE206785, and EAC samples from PRJNA777911 were included. GFRAL expression was largely low or undetectable across most cell populations and cohorts, with detectable expression mainly observed in selected non-malignant compartments in PRJNA777911.

**Supplemental table 1. Characteristics of the EAC patients for EAC CAFs included in this study.**

|  | TBE60 | TBE63 | CAF2304  (PDO2304) | CAF2765  (PDO2765) | CAF3095  (PDO3095) |
| --- | --- | --- | --- | --- | --- |
| **Age** | 50 | 57 | 81 | 70 | 65 |
| **Gender** | Female | Male | Male | Female | Male |
| **Sample source** | Biopsy | Biopsy | Operation | Operation | Operation |
| **Pathology** | EAC | EAC | EAC | EAC | EAC |
| **Treatment status at**  **sample collection** | Untreated  CROSS | Untreated  CROSS | Untreated | Untreated | Untreated |
| **Treatment strategy** | +surgery | +surgery | Surgery | Surgery | Surgery |
| **Neoadjuvant**  **therapy response** | Minor | Major | - | - | - |
| **TNM stage** | ypT3N2M0 | ypT3N1M0 | pT2N1M0 | pT3N2M0 | pT4aN3M0 |

**Supplemental table 2. Comparison of patient characteristics in serum GDF15 low group and high group before and after CROSS treatment.**

|  | Before CROSS (n=55) | | *P* | After CROSS (n=55) | | *P* |
| --- | --- | --- | --- | --- | --- | --- |
|  | GDF15 low  (n=28) | GDF15 high  (n=27) |  | GDF15 low  (n=33) | GDF15 high  (n=22) |  |
| **GDF15** (pg/ml), | 683.8 ± | 1202.7 ± | **<0.001** | 1237.1 ± | 2325.0 ± | **<0.001** |
| mean±SD | 154.6 | 274.4 |  | 260.5 | 754.1 |  |
| **Age** (years), mean ± SD | 61.5 ± 8.7 | 64.7 ± 9.4 | 0.461 | 62.0 ± 6.6 | 64.7 ± 12.2 | 0.302 |
| **Gender**, n (%) |  |  | 0.729 |  |  | 0.723 |
| Male | 22 (78.6) | 23 (85.2) |  | 26 (78.8) | 19 (86.4) |  |
| Female | 6 (21.4) | 4 (14.8) |  | 7 (21.2) | 3 (13.6) |  |
| **BMI** | 26.5 ± 4.5 | 28.4 ± 5.0 | 0.142 | 27.0 ± 4.5 | 28.1 ± 5.2 | 0.412 |
| **Response*** |  |  | 0.102 |  |  | 0.182 |
| Minor/Partial | 10 (37.0) | 16 (59.3) |  | 13 (40.6) | 13 (59.1) |  |
| Major/Complete | 17 (63.0) | 11 (40.7) |  | 19 (59.4) | 9 (40.9) |  |
| **ypT**, n (%) |  |  | 0.114 |  |  | 0.252 |
| T0-1 | 13 (46.4) | 7 (25.9) |  | 14 (42.4) | 6 (27.3) |  |
| T2-3 | 15 (53.6) | 20 (74.1) |  | 19 (57.6) | 16 (72.7) |  |
| **ypN**, n (%) |  |  | 0.891 |  |  | 0.322 |
| N0 | 14 (50.0) | 13 (48.1) |  | 18 (54.5) | 9 (40.9) |  |
| N1-3 | 14 (50.0) | 14 (51.9) |  | 15 (45.5) | 13 (59.1) |  |

*one missing data. Bold text indicates a significant difference.

**Supplemental table 3. Characteristics of the EAC patients with paired EAC tumor and adjacent normal tissue samples.**

| n=25 | |
| --- | --- |
| **Age** (years), mean±SD  **Gender**, n (%) Male  Female  **pT**, n (%) T1  T2  T3  T4  **pN**, n (%) N0  N1  N2  N3 | 73.5 ± 9.0  22 (88.0)  3 (12.0)  6 (24.0)  6 (24.0)  11 (44.0)  2 (8.0)  9 (36.0)  6 (24.0)  6 (24.0)  4 (16.0) |

**Supplemental table 4. List of primers. Gene Sequence (5’ to 3’)**

| GAPDH-for | GAAGGTGAAGGTCGGAGTC |
| --- | --- |
| GAPDH-rev | GAAGATGGTGATGGGATTTC |
| 18s rRNA-for | GCTTAATTTGACTCAACACGGGA |
| 18s rRNA-rev | AGCTATCAATCTGTCAATCCTGT |
| β-actin-for | AGAGCTACGAGCTGCCTGAC |
| β-actin-rev | AGCACTGTGTTGGCGTACAG |
| αSMA-for | TACATAGTGGTGCCCCCTGA |
| αSMA-rev | TTGCCTGATGGGCAAGTGAT |
| IL6-for | CCTGAACCTTCCAAAGATGGC |
| IL6-rev | TTCACCAGGCAAGTCTCCTCA |
| PDGFRα-for | TGGCAGTACCCCATGTCTGAA |
| PDGFRα-rev | CCAAGACCGTCACAAAAAGGC |
| Vimentin-for | ACGTCTTGACCTTGAACGCA |
| Vimentin-rev | CGTGAGGTCAGGCTTGGAAA |
| GDF15-for | GACCCTCAGAGTTGCACTCC |
| GDF15-rev | GCCTGGTTAGCAGGTCCTC |

**Key resources table**

| **REAGENT or RESOURCE** | **SOURCE** | **IDENTIFIER** |
| --- | --- | --- |
| **Antibodies** | | |
| Mouse monoclonal anti-α-Tubulin | Cell Signaling Technology | 3873 |
| Mouse monoclonal anti-GAPDH | Proteintech group | 60004-1-Ig |
| Mouse monoclonal anti-GDF15 | Sigma-Aldrich | AMAB90687 |
| Rabbit monoclonal anti-phospho- AKT (Ser473) | Cell Signaling Technology | 4058 |
| Rabbit monoclonal anti-phospho- AKT (Thr308) | Cell Signaling Technology | 13038 |
| Rabbit monoclonal anti-AKT (pan) | Cell Signaling Technology | 4685 |
| InVivoMAb Mouse IgG1 Isotype Control(MOPC-21) | AntibodySystem | VMJ92801 |
| InVivoMAb Mouse α-h-GDF15, 0297 |  | NA |
| Goat polyclonal anti-Mouse | Invitrogen | 31430 |
| Goat polyclonal anti-Rabbit | Invitrogen | 31460 |
| EnVision system-HRP anti- Rabbit | Dako North America | K4009 |
| EnVision system-HRP anti- Mouse | Dako North America | K4005 |
| Mayer’s hematoxylin solution | Panreac Quimica | 254766 |
| **Biological samples** | | |
| 25 paired EAC tumor and normal tissues | BIOMASOTA Biobank, University Hospital of Cologne | NA |
| 55 paired EAC patients’ serum samples before and after the CROSS treatment | BIOMASOTA Biobank, University Hospital of Cologne | NA |
| EAC tissue samples for cancer- associated fibroblasts and tumor organoids generation | BIOMASOTA Biobank, University Hospital of Cologne | NA |
| **Chemicals, peptides, and recombinant proteins** | | |
| Collagen type I | Corning | 354249 |
| Amphotericin B | R&D Systems | B23192 |
| Normocin | InvivoGen | ant-nr-2 |
| Fetal bovine serum (FBS) | Capricorn Scientific | FBS-12A |
| Penicillin/streptomycin (P/S) | Gibco | 15140-122 |
| DMEM/F-12 | Gibco | 12634-010 |
| L-Glutamine | Gibco | 25030-024 |
| DPBS | Gibco | 14190-094 |
| Dispase | Stem cell | 07913 |
| Collagenase IV | GENAXXON bioscience | c4310 |
| 0.25% Trypsin EDTA | Gibco | 25200056 |
| Matrigel matrix | Corning | 354230 |
| N-2 supplement | Life Technologies  Corporation | 17502048 |
| B-27 supplement | Life Technologies  Corporation | 17504001 |
| N-Acetyl-L-cysteine | Sigma-Aldrich | A9165 |
| N-Acetyl-L-cysteine | Sigma-Aldrich | A9165 |
| CHIR-99021 | MedChemExpress | HY-10182 |
| Recombinant Human EGF | PeproTech | AF-100-15 |
| Recombinant Human FGF-10 | PeproTech | 100-26 |
| A83-01 | Tocris Bioscience | 2939 |
| SB202190 | MedChemExpress | HY-10295 |
| [Leu15] Gastrin I human | Sigma-Aldrich | G9145 |
| Nicotinamide | Sigma-Aldrich | N0636 |
| Gentamicin | Life Technologies  Corporation | 15710064 |
| Recombinant Human Noggin | PeproTech | 120-10C |
| Y-27632 | Sigma-Aldrich | Y0503 |
| ROTI Histofix | Carl Roth | P807 |
| Crystal violet solution | Sigma-Aldrich | V5265 |
| Acetic acid | Sigma-Aldrich | A-6283 |
| MTT solution | Sigma-Aldrich | 475989 |
| Annexin V-APC | ImmunoTools | 31490016 |
| TRI reagent | Sigma-Aldrich | T9424 |
| NuPAGE LDS sample buffer | Life Technologies  Corporation | NP0007 |
| Roti-Block | Carl Roth | A151 |
| Triton X-100 | EuroClone | EMR237500 |
| Normal serum block | BioLegend | 927503 |
| Xylene | Sigma-Aldrich | X2377 |
| Ethanol | Carl Roth | 9065 |
| Citrate buffer | Carl Roth | 5110.3 |
| Hydrogen peroxide solution | Th. Geyer | 452.2511 |
| Methanol | Carl Roth | 8388.5 |
| Antibody diluent reagent solution | Life Technologies | 003218 |
| TBS buffer | Thermo Fisher Scientific | 28358 |

| Ampicillin | Carl Roth | HP62.2 |
| --- | --- | --- |
| Polyethylenimine (PEI) | Sigma-Aldrich | 408727 |
| Polybrene | MedChemExpress | HY-112735 |
| Puromycin | InvivoGen | ant-pr-1 |
| Human recombinant GDF15 protein | PeproTech | 120-28C |
| Cisplatin | Hexal AG | hH8716 |
| Oxaliplatin | Accord Healthcare | gy0776 |
| AKT inhibitor VIII | MedChemExpress | HY-10355 |
| Oligomycin A | AdipoGen | A01877/A |
| **Critical commercial assays** | | |
| MycoStrip kit | InvivoGen | rep-mys-50 |
| CellTiter-Glo Luminescent Cell Viability Assay | Promega | G7570 |
| High-Capacity cDNA Reverse Transcription Kit | Thermo Fisher Scientific | 4368814 |
| Fast SYBR green master mix | Thermo Fisher Scientific | 4385617 |
| BCA protein assay kit | Thermo Fisher Scientific | 2161296 |
| SuperSignal West Pico PLUS Chemiluminescent Substrate | Life Technologies | 34577 |
| Human GDF-15 Quantikine ELISA Kit | R&D Systems | DGD150 |
| Agilent Seahorse XF Cell Mito Stress Test Kit | Agilent Technologies | 103015 |
| QuantSeq 3' mRNA-Seq Library Prep Kit FWD for Illumina | Lexogen GmbH | NA |
| Zombie UV^TM^  Fixable Viability Kit | BioLegend | B482204 |
| TMRE, Tetramethylrhodamine ethyl ester, perchlorate | Promocell | PK-CA707-70016 |
| **Experimental models: Cell lines** | | |
| OE19 cell line | Sigma | 96071721 |
| OE33 cell line | Sigma | 96070808 |
| Cultrex HA-R-Spondin 1-Fc 293T cell line | R&D Systems | 3710-001-01 |
| HEK293T cell line | Odenthal group, University Hospital of Cologne | NA |
| Wnt-3a cell line | Hans Cleavers group, Hubrecht Institute | NA |
| TBE60 cell line, see Table S1 | Bruns/Zhao group,  University Hospital of Cologne | NA |
| TBE63 cell line, see Table S1 | Bruns/Zhao group,  University Hospital of Cologne | NA |

| CAF2304 cell line, see Table S1 | Bruns/Zhao group,  University Hospital of Cologne | NA |
| --- | --- | --- |
| CAF2765 cell line, see Table S1 | Bruns/Zhao group,  University Hospital of Cologne | NA |
| CAF3095 cell line, see Table S1 | Bruns/Zhao group,  University Hospital of Cologne | NA |
| PDO2304 organoids, see Table S1 | Bruns/Zhao group,  University Hospital of Cologne | NA |
| PDO2765 organoids, see Table S1 | Bruns/Zhao group,  University Hospital of Cologne | NA |
| PDO3095 organoids, see Table S1 | Bruns/Zhao group,  University Hospital of Cologne | NA |
| **Oligonucleotides** | | |
| Primer for GAPDH, see Table S4 | Thermo Fisher Scientific | NA |
| Primer for 18s rRNA, see Table S4 | Thermo Fisher Scientific | NA |
| Primer for β-actin, see Table S4 | Thermo Fisher Scientific | NA |
| Primer for αSMA, see Table S4 | Thermo Fisher Scientific | NA |
| Primer for IL6, see Table S4 | Thermo Fisher Scientific | NA |
| Primer for PDGFRα, see Table S4 | Thermo Fisher Scientific | NA |
| Primer for GAPDH, see Table S4 | Thermo Fisher Scientific | NA |
| sh-NT  5'-  AGGTAGTGTAATCGCCTTGTT-3' | Thermo Fisher Scientific | NA |
| sh-GDF15 #1  5'-  GCTCCAGACCTATGATGACTT-3' | Thermo Fisher Scientific | NA |
| sh-GDF15 #2  5'-  CCGGATACTCACGCCAGAAGT-3' | Thermo Fisher Scientific | NA |
| **Recombinant DNA** | | |
| pLKO.1 puro vector | Addgene plasmid | #8453 |
| psPAX2 | Addgene plasmid | #1226 |
| pMD2.G | Addgene plasmid | #12259 |
| **Software and algorithms** | | |
| ECHO pro, version 6.4.1 | ECHO | NA |
| GraphPad Prism, version 9.5.0 | GraphPad Software | NA |
| FlowJo, version 10.4 | BD Life Sciences | NA |
| Wave software, version <2.6.1.53> | Agilent Technologies | NA |

| STAR software, version 2.6 | <https://github.com/alexdob> in/STAR | NA |
| --- | --- | --- |
| ‘DESeq2’ R package, version 1.22.2 | 10.18129/B9.bioc.DESeq2 | NA |
| R software, version 3.5.3 | <https://www.r-project.org> | NA |
| MaxQuant, version 2.2 | <https://www.maxquant.org> | NA |
| ‘DEP’ R package, version 1.22.0 | 10.18129/B9.bioc.DEP | NA |
| IBM SPSS Statistics software, version 25 | IBM Corporation | NA |
| **Other** | | |
| BIOBEAM GM gamma irradiation device | Gamma Service Medical GmbH | NA |
| Attune ™ NxT Flow Cytometer | Life Technologies | NA |
| QuantStudio 7 Flex Real-Time PCR System | Life Technologies | 4485701 |
| Bioruptor Pico sonication system | Diagenode | NA |
| PVDF membrane | MACHEREY-NAGEL | 741260 |
| Trans-Blot Turbo transfer system | Bio-Rad Laboratories | NA |
| ChemoStar ECL Imager | INTAS Science Imaging | NA |
| Olympus IX83 inverted microscope | Evident Corporation | NA |
| PT-Module | Thermo Shandon Limited | NA |
| Seahorse XFe96 Analyzer | Agilent Technologies | NA |
| HiSeq 4000 system | Illumina | NA |
| ECHO Rebel microscope | ECHO | NA |
| Reagents for proteomics sample preparation | Proteomics Core Facility Cologne,  [www.proteomics.cecad](https://www.proteomics.cecad)- <labs.uni-koeln.de> | NA |
| Easy nanoLC 1000 system | Thermo Fisher Scientific | NA |
| The Genetic Perturbation Platform (GPP) | Broad Institute,  [https://portals.broadinstitute.or](https://www.ncbi.nlm.nih.gov/geo/query/acc.cgi?acc=GS) [g/gpp/public/](https://www.ncbi.nlm.nih.gov/geo/query/acc.cgi?acc=GS) | NA |
| The TCGA database,  (Esophageal Adenocarcinoma,  PanCancer Atlas) | [https://www.cbioportal.org/](https://www.ncbi.nlm.nih.gov/geo/query/acc.cgi?acc=GS) [study/summary?id=esca_tcga](https://www.ncbi.nlm.nih.gov/geo/query/acc.cgi?acc=GS) [_pan_can_atlas_2018](https://www.ncbi.nlm.nih.gov/geo/query/acc.cgi?acc=GS) | NA |
| GEPIA 2 | <http://gepia2.cancer>- <pku.cn/#index> | NA |
| GSE26886 database | [https://www.ncbi.nlm.nih.g](https://www.ncbi.nlm.nih.gov/geo/query/acc.cgi?acc=GS) [ov/geo/query/acc.cgi?acc=GS](https://www.ncbi.nlm.nih.gov/geo/query/acc.cgi?acc=GS) E26886 | NA |
| GSE92396 database | [https://www.ncbi.nlm.nih.g](https://www.ncbi.nlm.nih.gov/geo/query/acc.cgi?acc=GS) [ov/geo/query/acc.cgi?acc=GS](https://www.ncbi.nlm.nih.gov/geo/query/acc.cgi?acc=GS) E92396 | NA |
